# Layered Gradient Rhombic Dodecahedron Composite Structures for Biomimetic Bone Fabricated via Selective Laser Melting

**DOI:** 10.3390/mi16060673

**Published:** 2025-05-31

**Authors:** Yun Zhai, Tianyuan Zhong, Shuangquan Guo, Sheng Lin, David Hui, Xiaowei Ma

**Affiliations:** 1 School of Mechanical Engineering, Dalian Jiaotong University, Dalian 116028, China; yunzhai5@vip.163.com (Y.Z.); hgyumbdcla@126.com (T.Z.);; 2 State-Owned Chuanxi Machine Factory, Chengdu 611936, China; 3 Department of Mechanical Engineering, University of New Orleans, New Orleans, LA 70148, USA; 4 Department of Orthopedics, Affiliated Zhongshan Hospital of Dalian University, Dalian 116001, China

**Keywords:** biomimetic bone, gradient porous structure, stress transmission mechanism, selective laser melting, stress shielding

## Abstract

Porous bone implants have been extensively studied, with gradient structures receiving increasing attention due to their superior compatibility with bone tissue. However, comparative studies between gradient and uniform structures remain relatively scarce. In this study, selective laser melting (SLM) technology was employed to fabricate a gradient composite Ti6Al4V humeral bone plate, utilizing rhombic dodecahedron and its derived structures as unit cells. By adjusting the porosity parameter range to 22.02–94.37% using the Ashby Gibson formula, the mechanical properties of the porous bone plate were analyzed by varying the porosity parameters and conducting compression tests. The experimental results show that after preparing and compressing the structure, the elastic modulus of the model is controlled between 0.09–5.43 GPa, and the maximum yield strength is 216.1 Mpa. The experimental results demonstrate that, under shear loading, the gradient structure generates stress from the center of mass, with the phenomenon becoming more pronounced as the number of struts aligned with the direction of the applied load increases. This results in the model exhibiting characteristics of good resilience on the outside and a certain degree of rigidity on the inside. Compared to non-gradient models, gradient structures are more effective in controlling the direction of force transmission. Moreover, the elastic modulus of the bone plate is closer to that of natural bone tissue. These findings provide valuable insights for further research into gradient structure models of other rod-shaped unit cells, highlighting the mechanical advantages of gradient structures over uniform ones.

## 1. Introduction

With the continuous acceleration of population aging, there has been a sharp increase in the clinical demand for bone implants [1]. Among these, humeral shaft fractures account for approximately 5–8% of all fractures and 3% of all long bone fractures [2,3]. Currently, one of the major challenges faced by orthopedic implants is the stress shielding effect, which occurs when the significant difference in the elastic moduli between the implant and bone prevents the bone tissue from effectively bearing the load, leading to bone resorption [4,5,6,7]. The human skeleton exhibits a distinct gradient structure, and implants with a gradient design can better simulate the natural bone tissue, thereby enhancing the compatibility between the implant and the bone tissue [8]. Titanium alloy implants with a gradient porous structure can effectively alleviate the stress shielding phenomenon in the femur [9]. However, there is still a lack of understanding regarding the evolution mechanism of implant structure and macroscopic mechanical properties. This study aims to achieve optimal matching between the implant and bone tissue structure by gradient modification of unit cell arrays, coupled with a composite structural design.

Cortical bone is primarily located in the outer layer of long bones, where it functions to bear weight, provide support, and protect the bone marrow and cells. Its elastic modulus is approximately 17.5 GPa [10]. The thickness of the cortical bone in the humerus ranges from 0.33 to 3.5 mm [11]. Cancellous bone is located within the interior of the skeleton, responsible for maintaining the lightweight structure of bones, assisting in bone deformation, promoting vascular growth, as well as bone remodeling and metabolism. The elastic modulus of cancellous bone in the humerus ranges from 0.1 to 2 GPa [12]. Trabecular bone, serving as a transitional structure between cortical and cancellous bone, exhibits a variety of morphological variations, with a larger surface area and porosity. Its elastic modulus lies between that of cortical and cancellous bone, approximately ranging from 2 to 14 GPa [13]. The elastic modulus of titanium alloys is approximately 110 GPa [14]. Therefore, it is essential to reduce the elastic modulus of bone implants.

The most commonly used bone implant materials in clinical practice include bone cement and hydrogels. Although these materials exhibit good initial strength, they are prone to degradation after long-term implantation [15]. The application of metal implants primarily involves biocompatible alloys such as Ti6Al4V, CoCrMo, stainless steel, pure tantalum alloy, and NiTi alloys [16,17]. Titanium alloys have become the primary material of choice for orthopedic implants due to their excellent corrosion resistance, biocompatibility, antioxidative properties, and mechanical performance [18]. Feng et al. conducted two sets of control experiments, verifying that the porous humeral bone plate outperforms the solid titanium alloy plate in terms of postoperative recovery and effectiveness [19]. Liu et al. found that the attachment of tantalum (Ta) material to porous titanium bone plates significantly promoted bone growth in lamb legs and reduced the formation of calluses, further validating the positive impact of implant surface roughness on bone and vascular growth [20]. Lv et al. designed a radial gradient TPMS structure by adjusting parameters, demonstrating that the mechanical properties of Primitive unit cells are relatively superior under compression conditions. Additionally, the Primitive model exhibits good permeability in penetration testing [17]. Zhao et al.’s research indicates that pure magnesium screws, when used for fixation, show better results compared to traditional methods. Additionally, magnesium screws can dissolve in the body, releasing magnesium ions that further promote bone growth [21]. Dhandapani et al. designed biodegradable porous bone screws, which degrade into carbon dioxide and water, by controlling the printing infill density. They found that a pore size of 250–300 μm was most favorable for cell infiltration and capillary formation [22]. Zhu et al. found that TPMS with a porosity of 70% exhibits relatively good biocompatibility [23]. Xu et al. demonstrated that the postoperative recovery effect of porous tantalum coated with magnesium-doped calcium phosphate, prepared using SLM, was superior to that of pure porous tantalum [24]. Bai et al. utilized surface modification techniques to promote the growth of osteocytes and enhance the biocompatibility of implants [25]. Yao et al. prepared three different gradient types for Primitive unit cells using SLS. Among them, the linear gradient exhibited relatively good mechanical properties and high permeability, making it effective for customizing gradient porous scaffolds with specific properties [26]. Sadlik et al. studied titanium alloy and hydroxyapatite (HAp) composite materials, developing a biomaterial that promotes bone growth and effectively reduces the issue of implant loosening [27]. Chen et al. summarized that the application of antibacterial coatings on implants significantly reduces the incidence of associated infections [28]. These studies have driven the structural optimization of implants, reduced the stress-shielding effect, and further enhanced their compatibility with bone tissue.

The geometric shape of the unit cell has a significant impact on the mechanical properties of bone implants. Cheng et al. fabricated porous tantalum diamond structures and porous Ti6Al4V rhombic dodecahedron structures using SLM technology, demonstrating that the porous Ti6Al4V scaffold with a rhombic dodecahedron structure not only possesses high mechanical strength but also exhibits relatively good osteogenic performance [29]. Munyensanga et al. studied CoCrMo alloys and found that the rhombic dodecahedron structure exhibited excellent yield stress (9.36 MPa) and corrosion resistance [30]. Zhai et al. conducted a study on models of octahedral and rhombic dodecahedron structures with different sizes and determined that when the edge length of the octahedron is 1.5 mm and the strut width is 0.4 mm, the mechanical properties and elastic modulus of the model are closest to those of human bone [31]. Ziaie et al. achieved a higher strain-energy density ratio by fabricating a triply-period minimal surface structure [32]. Ni et al. prepared a triply-periodic minimal surface and rhombic dodecahedron (RDOD) with the same porosity for yield strength comparison. The yield strength of the TPMS scaffold with 70% porosity was 30% higher than that of RDOD with the same porosity, and its elastic modulus also exceeded the theoretical range of human trabecular bone [33]. Ziaie et al. reduced the yield strength and ultimate tensile strength by preparing Gyroid and Diamond models followed by heat treatment, while significantly increasing the elongation, thereby enhancing the isotropy of the porous design structures [34]. These studies have validated the feasibility and tremendous potential of the rhombic dodecahedron structure in bone implant design.

In recent years, with the rapid development of additive manufacturing (AM) technology, many emerging techniques have been derived, such as selective laser melting (SLM), electron beam melting (EBM), direct energy deposition (DED), fused filament fabrication (FFF), and selective laser sintering (SLS). These techniques can be used to manufacture porous structures and implants with complex surfaces [35]. These implants can be customized based on the individual injury conditions of the patient, and, compared to traditional methods, they allow for precise adjustment of the material composition and structure in three-dimensional space [36]. Xu et al. prepared a gradient structure model of PLA and TTCP composite materials with gyro unit cells using SLS technology. Through three types of dimensional gradient processing, a gradient structure model centered at the origin was obtained, exhibiting advantages in compressive strength and permeability [37]. Wu et al. prepared topologically optimized structures and rhombic dodecahedron structures using LPBF, and found that the topologically optimized structures exhibit significant compressive fatigue durability [38]. Dong et al. prepared ten types of TPMS aluminum alloy materials using SLM technology and found that Diamond, F-RD (r), Costa, and F-KC (Y) porous structures exhibit higher hydrogen production rates and shorter induction times, indicating that TPMS structures (such as surface area and pore throat size) significantly influence hydrogen production performance [39]. Cui et al. fabricated porous titanium alloy structures using SLM technology, adjusting the unit cell parameters to reduce the elastic modulus to 0.74 GPa, while achieving excellent yield strength (201.91 MPa). Among these, the dodecahedron structure exhibited optimal mechanical properties [40]. Liu et al. fabricated porous Ti6Al4V using selective laser melting (SLM) technology, with a measured elastic modulus of 28.78 GPa and a density of 4172 kg/m^3^ [41]. These achievements are all attributed to the rapid development of additive manufacturing technology.

**Figure 1:**
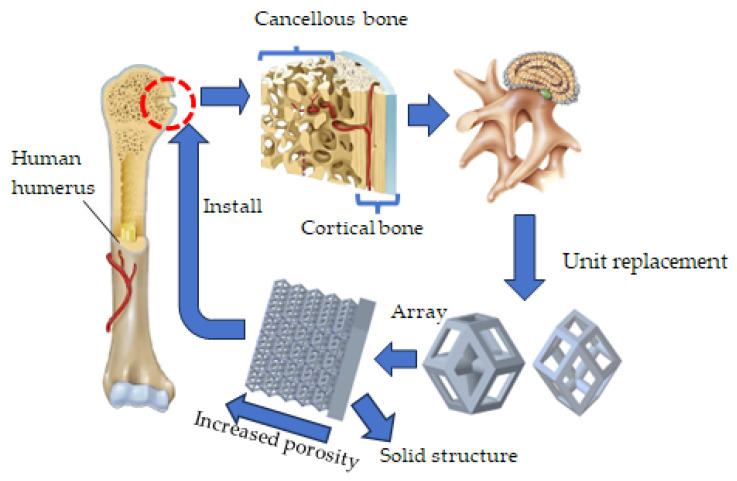
The preparation process of humeral bone plate.

Despite the widespread progress in research on alloy implants, the issue of achieving perfect compatibility between bone tissue and implants has yet to be fully addressed. This study starts from the structure and mechanical properties of the humerus and humeral bone plates and constructs a segmented gradient model based on the rhombic dodecahedron and its derivative structures. The solid part in the composite structure is used to match cortical bone, while the gradient porous structure is used for biomimetic cancellous bone. The technical route is shown in Figure 1. Combined with a solid composite structure, a titanium alloy bone plate was fabricated using SLM technology. The mechanical conduction mechanism of the gradient implant was elucidated, providing a theoretical basis for further optimizing the compatibility between alloy implants and human bone tissue.

## 2. Materials and Methods

### 2.1. Structural Design

#### 2.1.1. Selection of Unit Cells and Gradient Structure Design

**Figure 2:**
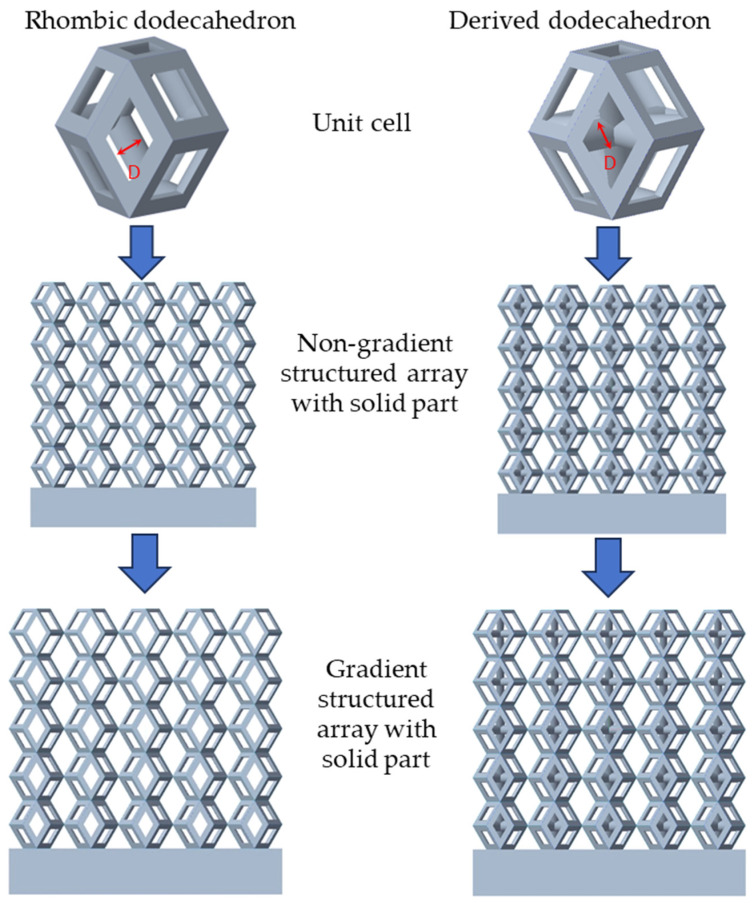
The flowchart of model establishment.

There are various types of unit cells in porous structures, and these structural variations significantly influence the mechanical properties of materials, such as ductility, elastic modulus, conductivity, thermal conductivity, biocompatibility, cell adhesion, and corrosion resistance. By selecting the optimal unit cell and fine-tuning the material’s structure, its performance can be enhanced, making it better suited for practical applications [42]. A rhombic dodecahedron structure, in particular, offers distinct advantages in terms of biocompatibility and cell adhesion, owing to its high porosity and specific surface area. The choice of unit cell also plays a critical role in reducing the anisotropy of the elastic modulus while simultaneously improving mechanical properties, thereby achieving a closer match in elastic modulus with bone tissue. The rhombic dodecahedron has a relatively low elastic modulus and a relatively high porosity, which can avoid stress shielding after implantation of biomimetic bone implants, prolong the service life of implants, and prevent sterile loosening caused by structural failure of implants after long-term implantation. In this study, two-unit cell models—namely the rhombic dodecahedron and a derived dodecahedron—are selected for optimization based on previous research, as illustrated in Figure 2. The modeling software used in this project is Creo Parametric 7.0. By arranging the unit cells along the three principal axes (XYZ), a porous structure model was generated. This array method prepares for gradient processing of the *Z*-axis unit cells of the model in the future, ensuring that only the *Z*-axis is variable and the rest are isotropic. The centroid spacing between the unit cells of the rhombic dodecahedron and its derivative dodecahedron along the perpendicular to the direction of the structural gradient of layered structure composed of two types of unit cells is 2.95 mm, which is 0.05 mm less than the distance between the two vertices of the unit cell. This slight reduction ensures that the unit cells can make contact perpendicular to the direction of the structural gradient of layered structure composed of two types of unit cells, preventing significant stress concentration that could arise from point-to-point contact. As mentioned earlier, long bones consist of cortical and cancellous bones. Cortical bone, typically solid, functions as the primary load-bearing component and has a thickness ranging from 0.33 to 3.5 mm [11]. To better mimic natural bone tissue, the experimental model in this study incorporates solid structures of varying thickness beneath the porous scaffold. Additionally, to more accurately replicate the biological properties of cancellous bone, gradient treatment was applied to both porous structures.

**Figure 3:**
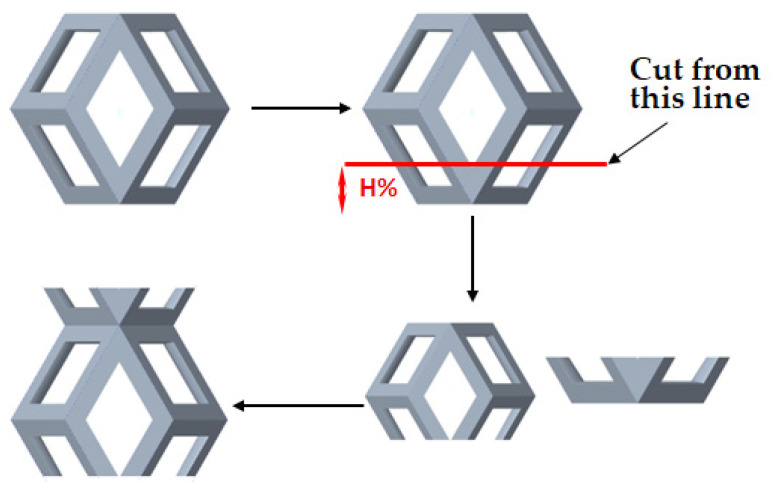
Model cutting method.

As shown in Figure 3, the unit cell is sectioned along its height, with H% defined as the ratio of the distance from the base to the cutting plane relative to the total height of the unit cell. The cut portion at the bottom is relocated to the top of the unit cell to form a new unit cell, ensuring that both the height and the equivalent volume of the new unit cell remain unchanged. By applying array and gradient processing to the unit cell, an entirely new porous structure can be generated. This transformation alters the contact area between the porous and solid structures while maintaining the overall equivalent volume of the model. Consequently, it enables the analysis of the relationship between the contact area and the shear elastic modulus. We assign H% values of 20%, 40%, 50%, 60%, and 80%, respectively. As shown in Table 1, the red region indicates the contact area between the porous structure and the solid structure after cutting and gradient-based arraying. It can be observed that the contact interface exhibits varying contact patterns and areas at different heights of the unit cell.

#### 2.1.2. Calculation of Porosity Parameters

The porosity of porous structures is an important parameter in model construction. However, due to the significant differences in the characteristics of porous structures, the mechanical properties of porous structure arrays will exhibit different presentations. Here, referring to the G-A formula proposed by Ashby and Gibson in 1997 [43]:

(1) $$\frac{E^{*}}{E}={\left(1-\emptyset_{P}\right)}^{2}$$

In Equation (1), E* represents the equivalent elastic modulus, E is the material’s elastic modulus, and $\emptyset_{P}$ denotes the equivalent porosity. As previously mentioned, the long bones of the human body consist of cortical bone and cancellous bone. The primary function of cortical bone is load-bearing, with a thickness ranging from 0.33 to 3.5 mm and a tensile and compressive elastic modulus of approximately 17.5 GPa. In contrast, the elastic modulus of cancellous bone is relatively low, typically ranging from 0.1 to 5 GPa. Due to the continuous metabolic processes and adaptive variations in bones with respect to time, these values can fluctuate and are influenced by factors such as age, gender, bone location, and mineralization level. Therefore, the range of elastic modulus used for studying pore structure is set between 0.1 and 17.5 GPa. Based on the G-A equation, the corresponding porosity range is calculated to be between 94.37% and 22.02%.

The model parameters for different unit cell arrays vary significantly, and porosity is primarily controlled by adjusting the edge length and edge diameter. For the two types of cell arrays, after multiple iterations, this study uses a unified edge length of 1.5 mm and a variable edge diameter D for both models. This approach allows for more precise control of porosity by modifying the diameter. Through continuous adjustments, the diameter range is set between 0.3 mm and 0.7 mm, with increments of 0.05 mm.

In the model parallel to the direction of the structural gradient of layered structure composed of two types of unit cells, the gradient structures of both models are created by reducing the edge diameter of each layer parallel to the direction of the structural gradient of layered structure composed of two types of unit cells by 0.05 mm, while maintaining the edge diameter values consistent with the non-gradient model, ranging from 0.3 mm to 0.7 mm. To ensure consistency with the non-gradient model, the gradient model also consists of 5 layers. The model codes are defined as follows: models with edge diameters ranging from 0.3 mm to 0.5 mm are designated as A1, from 0.35 mm to 0.55 mm as A2, from 0.4 mm to 0.6 mm as A3, from 0.45 mm to 0.65 mm are coded as A4, and those from 0.5 mm to 0.7 mm as A5. The porosity of each layer within the porous structure varies, and thus the model alters the porosity interval span of the porous structure and integrates it into an equivalent porosity. This directly influences the macroscopic mechanical properties of the model. When combined with the solid structure, the layered linear gradient porous architecture enables the biomimetic bone to achieve structural and elastic modulus compatibility with the humerus.

**Figure 4:**
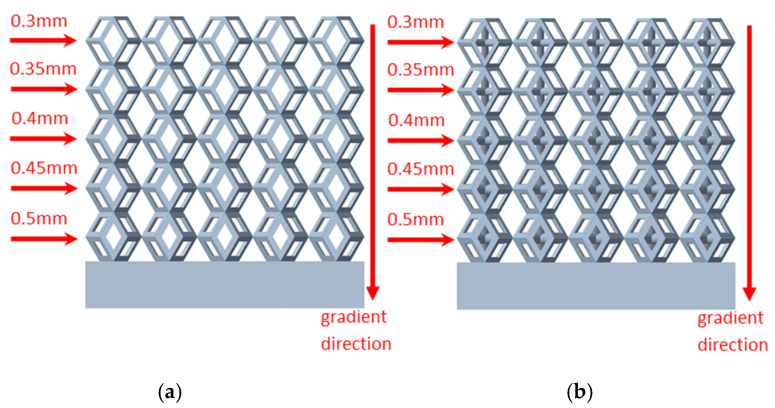
Gradient structures of two types of unit cells. (**a**) Rhombic Dodecahedron as A1; (**b**) Derived Dodecahedron coded as A1.

Figure 4a shows the rhombic dodecahedron model coded as A1, while Figure 4b shows the derived dodecahedron model coded as A1.

**Figure 5:**
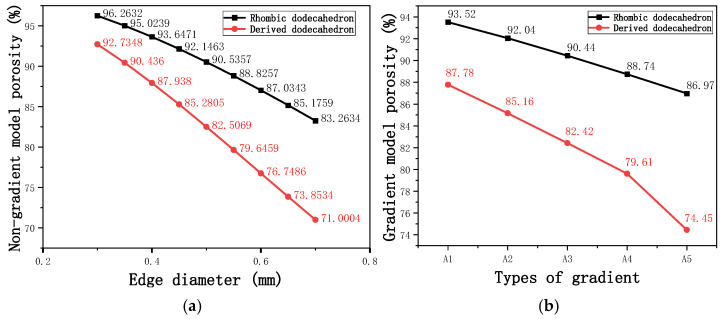
Gradient and non-gradient model porosity. (**a**) Porosity of non-gradient model with respect to edge diameter of unit cells; (**b**) Porosity of gradient model with respect to types of gradient of unit cells.

By modeling the unit cell and calculating the porosity after array formation using Creo, the variation in porosity for both non-gradient and gradient models can be analyzed using Creo’s built-in edge diameter volume calculation tool, as shown in Figure 5a,b.

Based on the data from the line chart, we can conclude that the porosity range of the non-gradient model is larger than that of the gradient model. Both the non-gradient and gradient models show more significant variations in the porosity of the derived dodecahedron, while the variation in the porosity of the rhombic dodecahedron is less pronounced. This can be attributed to the internal structure of the unit cell in both models, which results in the derived dodecahedron having more trusses than the rhombic dodecahedron. Consequently, compared to the rhombic dodecahedron, the porosity is more sensitive to variations in edge diameter, leading to a greater variation in porosity.

### 2.2. Preparation of One-Dimensional Gradient Porous Structures

**Figure 6:**
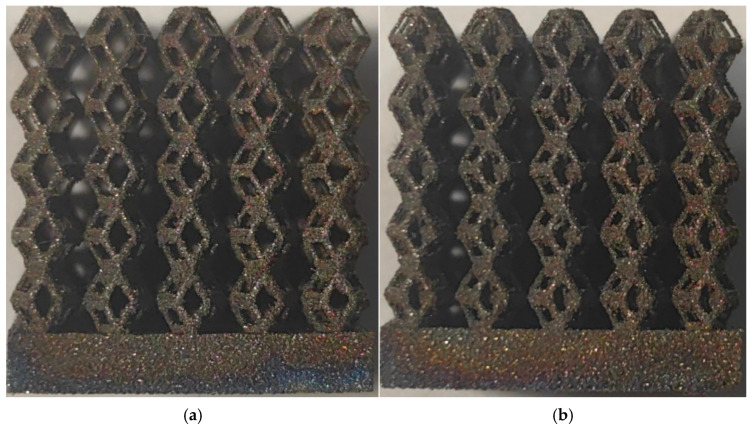
The forming effect of two models without height cutting. (**a**) The effect of forming rhombic dodecahedron gradient structure with SLM; (**b**) The effect of forming derived dodecahedron gradient structure with SLM.

The 3D printing equipment used in this study is the AM250 laser selective melting metal 3D printer produced by Renishaw in the UK. Its scanning spacing is 0.1 mm, laser power is 180 W, substrate temperature is 40 °C, printing layer height is 30 μm, exposure time is 50 μs, scanning speed is 1000 mm/s, and laser energy density is 269.1 J/mm^3^. The material is Ti6Al4V, and the material composition is shown in Table 2. The obtained samples are shown in Figure 6 and Table 3.

By increasing the observation scale of the gradient porous structure, as shown in Figure 6a,b, a noticeable variation in porosity can be observed from the solid base to the top. This confirms that the printed results of the two models are consistent with the simulation results, exhibiting a stepped gradient in porosity. Table 3 shows two perspectives of different cutting heights of two unit cells, and the shape of the structure can be clearly seen in the table.

### 2.3. Experimental Characterization

#### 2.3.1. Appearance Observation of Porous Structure

The scanning electron microscope (SEM) is an observation technique that lies between the transmission electron microscope and optical microscope in terms of resolution. It uses a focused, narrow high-energy electron beam to scan the sample. In this study, a JSM-6360LV scanning electron microscope, manufactured by JEOL USA, Inc. (Peabody, MA, USA), was used to observe the individual structures in detail after printing.

#### 2.3.2. Compression Measurement

Compression testing of porous structures is a crucial step in evaluating the reasonableness of the overall structural design and stress distribution. This process allows verification of the structure’s mechanical properties under external load, including compressive strength, flexural strength, shear strength, and deformation characteristics, ensuring consistency with simulation results. These tests validate the effectiveness of the simulation and confirm the model’s stability and reliability prior to implantation into the human body. The compression testing in this study was conducted using a UTM5105 electronic universal testing machine, which features an accuracy level of 0.5, a maximum compression force of 100 KN, a power rating of 1.5 kW, a working voltage of 380 V, and a compression speed of 5 mm/min.

### 2.4. Finite Element Meshing and Boundary Conditions

The finite element simulation software used in this study is ANSYS (version 2022 R1), utilizing the static structural module. In the engineering data settings, the material properties are defined with a density of 4172 kg/m^3^, Young’s modulus of 28.78 GPa, and a Poisson’s ratio of 0.3 under isotropic elasticity. The material is assigned as elastic. The grid size is set to 0.1 mm, and a tetrahedral grid analysis method is employed. Using finite element analysis, a displacement of 10% is applied under structural compression conditions to ensure the accuracy of the simulation results [44].

#### 2.4.1. Boundary Conditions for the Compression Perpendicular to the Direction of Structural Gradient of Layered Structure

**Figure 7:**
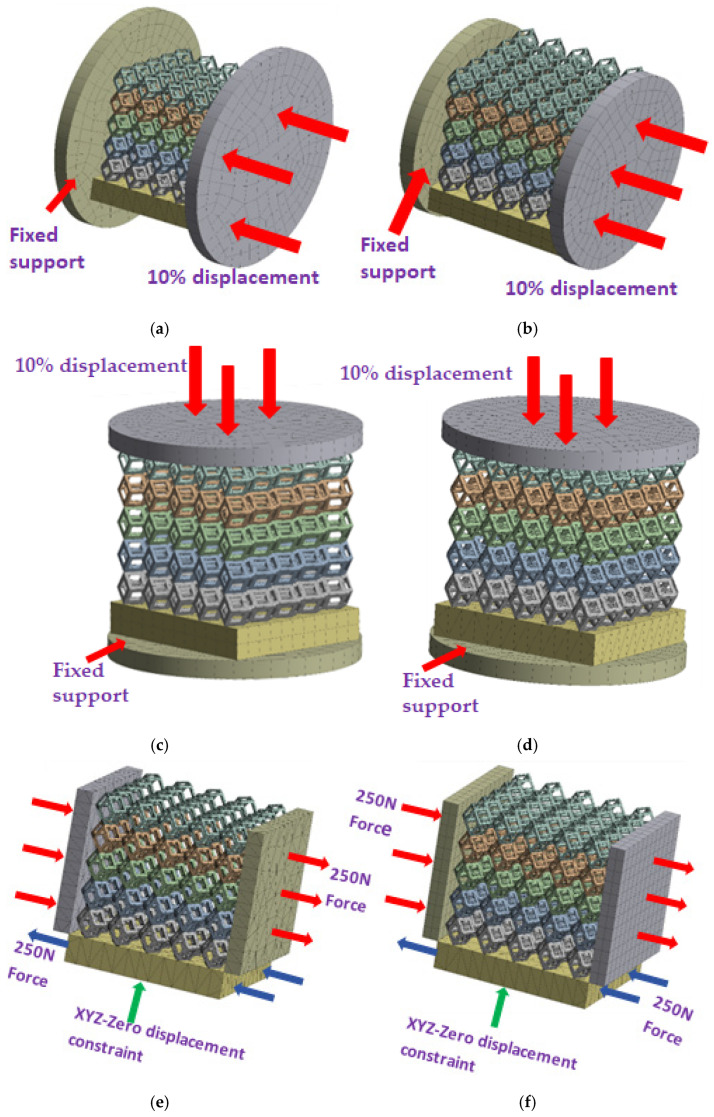
Boundary condition settings for three operating conditions of two structures. (**a**) Boundary conditions for perpendicular compression of the rhombic dodecahedron gradient model; (**b**) Boundary conditions for perpendicular compression of the derived dodecahedron gradient model; (**c**) Boundary conditions for directional compression of the rhombic dodecahedron gradient model; (**d**) Boundary conditions for directional compression of the derived dodecahedron gradient model; (**e**) Boundary conditions for shear of the rhombic dodecahedron model; (**f**) Boundary conditions for shear of the derived dodecahedron model.

To simulate the stability and reliability of porous structures perpendicular to the direction of the structural gradient of layered structure composed of two types of unit cells, ANSYS Static Structural module was used for compression simulations in this orientation. As described in Section 2.4, the overall model was meshed with a grid size of 0.1 mm. A displacement of 10% was applied to 50 vertices on the left side of the rhombic dodecahedron model and the left plane of the solid region. Fixed supports were assigned to 50 points on the opposite side and the right plane of the solid region. The same setup was applied to the derived dodecahedron model, as illustrated in Figure 7a,b.

#### 2.4.2. Boundary Conditions for the Compression Parallel to the Direction of the Structural Gradient of Layered Structure

Compression simulation parallel to the direction of the structural gradient of layered structure composed of two types of unit cells was performed using the ANSYS Static Structural module. As outlined in Section 2.4, the entire model was meshed with a grid size of 0.1 mm. A displacement of 10% was applied to the top plane parallel to the direction of the structural gradient of layered structure composed of two types of unit cells, while the bottom plane was assigned as a fixed support, as illustrated in Figure 7c,d.

#### 2.4.3. Boundary Conditions for Shear Stress Acting on the Interface Between the Porous and Solid

When two materials with a significant difference in elastic modulus come into contact, stress concentration typically occurs at the contact surface. Therefore, it is essential to analyze the shear force in the model. As shown in Figure 7e,f, the boundary conditions for the shear direction of the two models are set. To make the simulation conditions as realistic as possible, a force of 250 N is applied to the 50 vertices on the left and 50 vertices on the right of the porous section of the rhombic dodecahedron, simulating the pressure exerted by the fixture on the model. A 250 N force is applied to the left and right planes of the solid section, totaling 500 N [45], with the two forces being opposite and parallel. The bottom plane is subjected to displacement constraints, with a displacement of 0 mm in the X, Y, and Z directions. The mesh is divided into tetrahedral elements with a size of 0.1 mm, consistent with Section 2.4.

## 3. Results and Discussion

### 3.1. Microstructure Characterization

**Figure 8:**
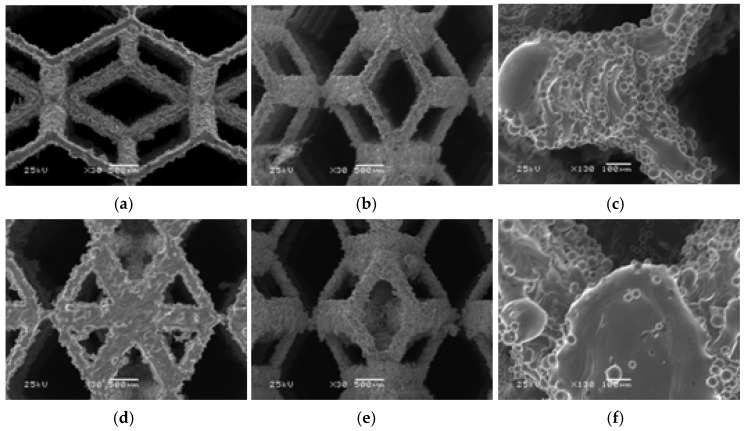
The morphology of structures of rhombic dodecahedron and its derivative processed by SLM technology. (**a**) Top view of rhombic dodecahedron at 30× magnification; (**b**) Front view of rhombic dodecahedron at 30× magnification; (**c**) Rhombic dodecahedron edge nodes at 130× magnification; (**d**) Top view of derived dodecahedron at 30x magnification; (**e**) Front view of derived dodecahedron at 30× magnification; (**f**) Derived dodecahedron edge nodes at 130× magnification.

Figure 8a–c depict the morphology, pore size, and microstructure of the rhombic dodecahedron as observed via scanning electron microscopy (SEM). Figure 8d–f illustrate the morphology, pore size, and microstructure of the derived dodecahedron as observed via scanning electron microscopy. The images clearly show that the surface of the struts in the metal scaffold is typically uneven, with particles adhering to it, while the connections between struts exhibit good integrity without obvious fractures. Additionally, the connections between unit cells are well connected. This phenomenon arises from the incomplete melting of titanium alloy particles, which adhere to the strut surface, thereby increasing its surface roughness. This enhanced roughness promotes the adhesion of biological cells to the struts, facilitating cell differentiation and bone regeneration [46].

### 3.2. Simulation Result Analysis

#### 3.2.1. The Compression Parallel to the Direction of Structural Gradient of Layered Structure Composed of Two Types of Unit Cells

In the simulation, we calculate the stress and strain by setting the displacement and solving the reaction force:

(2) $$\sigma =\frac{F_{re}}{S}$$

(3) $$\varepsilon =\frac{∆L}{L}$$

(4) $$E=\frac{\sigma }{\varepsilon }$$

Among them, *σ*, $F_{re}$, *S*, *ε*, ∆L, *L*, *E* represents the stress (MPa), support reaction force (N), equivalent area during compression (mm^2^, not the actual compression contacts area), actual displacement (mm), the original height of the porous structure in the compression direction (mm), elastic modulus.

**Figure 9:**
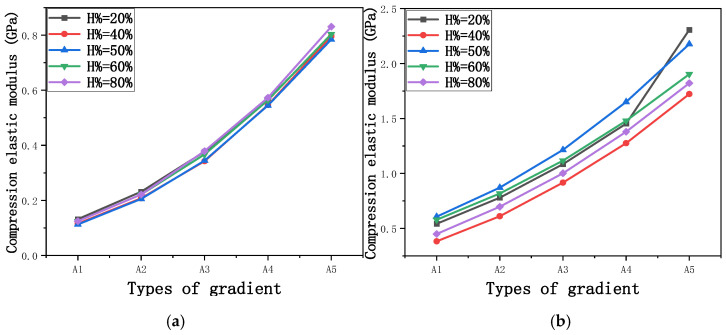
Compression elastic modulus parallel to the direction of the structural gradient of layered structure composed of two types of unit cells. (**a**) Compression elastic modulus parallel to the direction of the structural gradient of layered structure composed of rhombic dodecahedron; (**b**) Compression elastic modulus parallel to the direction of the structural gradient of layered structure composed of derived dodecahedron.

The equivalent elastic modulus of the two models obtained through Equation (4) are shown in Figure 9.

As shown in Figure 9, no significant difference is observed in the equivalent elastic modulus parallel to the direction of the structural gradient of the porous structure formed by the cut unit cell array. The compressive elastic modulus of the rhombic dodecahedron parallel to the direction of the structural gradient of layered structure composed of two types of rhombic dodecahedron, ranges from 0.1131 to 0.5127 GPa, while the compressive elastic modulus of the derived dodecahedron spans from 0.3827 to 2.3107 GPa. Both structures exhibit compatibility with the equivalent elastic modulus of the humerus. Among these, both the magnitude and range of the elastic modulus of the derived dodecahedron exceed those of the rhombic dodecahedron. This is attributed to the internal truss structure of the derived dodecahedron, which provides enhanced resistance to compression along the direction of the structural gradient of layered structure composed of derived dodecahedron. Additionally, the elastic modulus of the derived dodecahedron demonstrates greater sensitivity to increases in radial width. In contrast, the rhombic dodecahedron lacks struts oriented parallel to the direction of the structural gradient of layered structure composed of two types of rhombic dodecahedron, which significantly reduces its compressive resistance. Therefore, the elastic modulus of the rhombic dodecahedron is lower than that of the derived dodecahedron. Table 4 presents the stress distributions of rhombic dodecahedron and its derivation, with and without gradient with respect to time, when the direction of compressive stress is parallel to the gradient direction. The compression stroke is 10% of the total height, and the simulation time is 1 s. The stress distributions are displayed at the beginning of compression (0 s), and at 0.25 s, 0.5 s, 0.75 s, and 1 s. As mentioned in Figure 9, cutting the model height has a negligible effect on the elastic modulus of the model itself. Therefore, the model code A1 was used to maintain consistent porosity and edge diameter variables when extracting the stress distributions. From the table, it is evident that during the compression process, the non-gradient model undergoes significant deformation in the direction perpendicular to the structural gradient of layered structure composed of two types of unit cells. Consequently, the stress transmission is relatively dispersed, hindering accurate stress transfer to the solid structures. In contrast, the gradient structure exhibits a more controlled stress transmission direction, thereby reducing the risk of damage to bone tissue caused by uncontrolled stress propagation.

#### 3.2.2. The Compression Perpendicular to the Direction of Structural Gradient of Layered Structure Composed of Two Types of Unit Cells

**Figure 10:**
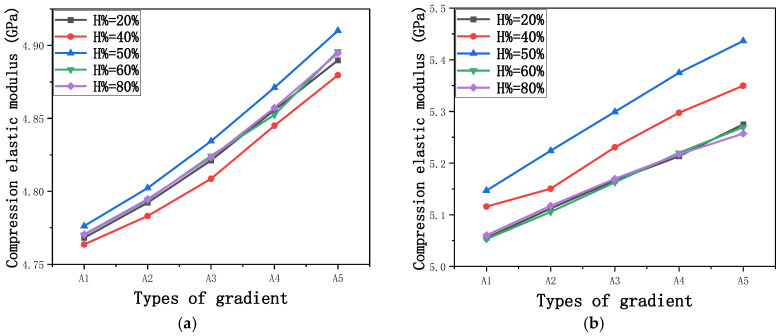
Compression elastic modulus perpendicular to the direction of the structural gradient of layered structure composed of two types of unit cells. (**a**) Compression elastic modulus perpendicular to the direction of the structural gradient of layered structure composed of rhombic dodecahedron; (**b**) Compression elastic modulus perpendicular to the direction of the structural gradient of layered structure composed of derived dodecahedron.

Figure 10 shows the equivalent compressive elastic modulus perpendicular to the direction of structural gradient of layered structure composed of two types of unit cells. Similar to the compressive elastic modulus parallel to the direction of the structural gradient of layered structure composed of two types of unit cells., the difference in elastic modulus between the models after height cutting is negligible. The gradient perpendicular elastic modulus of the rhombic dodecahedron ranges from 4.7636 to 4.9101 GPa, while the gradient perpendicular elastic modulus of the derived dodecahedron ranges from 5.0531 to 5.4365 GPa. Both structures exhibit compatibility with the equivalent elastic modulus of the humerus, and the difference in the magnitude of their elastic modulus ranges is consistent with the results presented in Section 3.2.1, which will not be reiterated here. Compression elastic modulus perpendicular to the direction of the structural gradient of layered structure composed of two types of unit cells. (a) Compression elastic modulus perpendicular to the direction of the structural gradient of layered structure composed of rhombic dodecahedron; (b) Compression elastic modulus perpendicular to the direction of the structural gradient of layered structure composed of derived dodecahedron.

Table 5 presents the stress distributions of both models, with and without gradient with respect to time, perpendicular to the direction of structural gradient of layered structure composed of two types of unit cells. The compression stroke is 10% of the structure height, and the stress distribution maps are shown at the following time intervals: 0 s (beginning of compression), 0.25 s, 0.5 s, 0.75 s, and 1 s (end of compression). Figure 10 demonstrates that cutting the unit cell height has negligible effects on the elastic modulus of the porous structure itself. Therefore, when extracting the stress distribution maps, model code A1 was used to consistently control the variables of porosity and edge diameter. From the table, it can be observed that regardless of whether the structure is a rhombic dodecahedron or a derived dodecahedron, their non-gradient models exhibit almost no stress variation in the strut perpendicular to the compression displacement direction, i.e., parallel to the direction of structural gradient of layered structure composed of two types of unit cells. In contrast, the gradient structure model exhibits full stress along the strut diameter parallel to the direction of the structural gradient of layered structure composed of two types of unit cells., demonstrating superior mechanical performance compared to the non-gradient structure model. This more reasonable stress distribution, along with smaller maximum stress, can extend the implant’s in vivo implantation time and prevent structural failure during bone tissue recovery. As anticipated, both the rhombic dodecahedron and its derivative exhibit greater stress in the lower porosity and solid structure regions [13], with lower porosity leading to higher stress, consistent with the initial design intent. The solid part functions similarly to cortical bone, primarily serving a load-bearing role.

**Figure 11:**
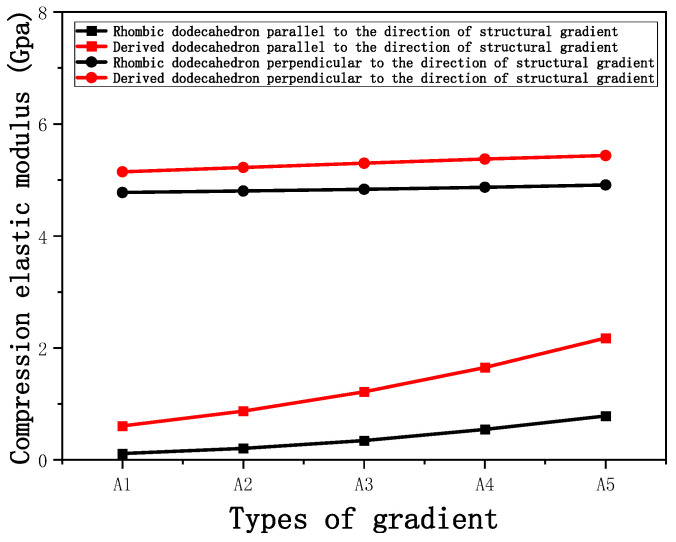
Compression elastic modulus parallel and perpendicular to the direction of the structural gradient of layered structure composed of two types of unit cells. of layered structure composed of layered structure composed of two types of unit cells.

Figure 11 shows the trends of porosity variation with the elastic modulus parallel to the direction of structural gradient of layered structure composed of two types of unit cells and perpendicular to the direction of structural gradient of layered structure composed of two types of unit cells. Circular points represent the elastic modulus perpendicular to the direction of structural gradient of layered structure composed of two types of unit cells, while square points represent the elastic modulus parallel to the direction of structural gradient of layered structure composed of two types of unit cells. It can be observed that the elastic modulus perpendicular to the direction of structural gradient of layered structure composed of two types of unit cells. is greater than that parallel to the direction of structural gradient of layered structure composed of two types of unit cells. for both models. However, the elastic modulus parallel to the direction of structural gradient of layered structure composed of two types of unit cells is more sensitive to the increase in edge diameter, i.e., the decrease in porosity. This is because the elastic modulus perpendicular to the direction of structural gradient of layered structure composed of two types of unit cells primarily relies on the solid part for load-bearing, whereas the elastic modulus parallel to the direction of structural gradient of layered structure composed of two types of unit cells is mainly supported by the struts. As a result, the elastic modulus parallel to the direction of structural gradient of layered structure composed of two types of unit cells is more sensitive to variations in edge diameter, i.e., variations in porosity. This phenomenon aligns with the initial intention of gradient and solid part designs, where the solid part and thicker struts sections are responsible for load-bearing, while regions with lower porosity are responsible for coordinating deformation and force transmission.

#### 3.2.3. Shear Force Acts on the Interface Between the Porous and Solid

As mentioned in Section 2.4.3, when two materials with a significant difference in elastic modulus come into contact, stress concentration will occur at the contact surface. The elastic modulus of the solid structure in this article is 28.78 GPa, while the elastic modulus of the porous structure ranges from 0.1 to 17.5 GPa. Therefore, there will always be stress concentration at the contact surface when stress is generated. According to the formula:

(5) $$G=\frac{\tau }{\gamma }$$

(6) $$\tau =\frac{F}{S}$$

(7) γ=tany

Among them, *G*, *τ*, *γ*, *F*, *S*, and *y* are shear elastic modulus, shear stress, shear strain, shear force, cross-sectional area of the shear plane, and angle after deformation, respectively. As mentioned earlier, the model was highly cut, and the contact area after cutting changed. We used the ratio of the contact area between the porous structure and the solid part to the area of the solid part as the independent variable to explore the effect of changing the ratio on the shear modulus. Given the definition of ratio as a Ratio, the larger the Ratio, the larger the cross-sectional area of the porous structure. The shear elastic modulus G and ratio of the two models are shown in Table 6. From Table 6, it can be concluded that the shear elastic modulus of the rhombic dodecahedron is 0.033 GPa–0.3876 GPa, and the shear elastic modulus of the derived dodecahedron is 0.1454 GPa–0.5794 GPa. The shear elastic modulus of the derived dodecahedron is the highest, while that of the rhombic dodecahedron is the lowest. The range of elastic modulus is consistent with the range of elastic modulus parallel to the direction of structural gradient of layered structure composed of two types of unit cells. and perpendicular to the direction of structural gradient of layered structure composed of two types of unit cells. The derived dodecahedron is greater than the rhombic dodecahedron, for the reasons mentioned earlier. From Table 6, it can be seen that the ratios of 20% and 80% height cuts, as well as the ratios of 40% and 60% height cuts, are almost the same in the rhombic dodecahedron and derived dodecahedron models. However, the ratio of 50% height cuts is much higher than that of other height cuts. This is because the inherent properties of the unit cells of the rhombic dodecahedron and derived dodecahedron determine that the two cell models are vertically symmetrical, with the largest edge diameter at 50%, so the ratio will be much higher than that of other heights cuts.

**Figure 12:**
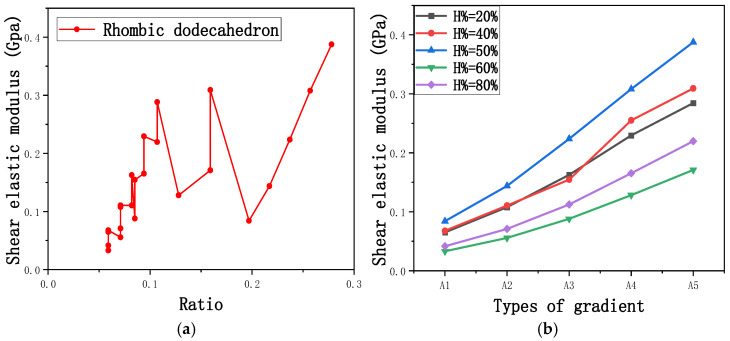

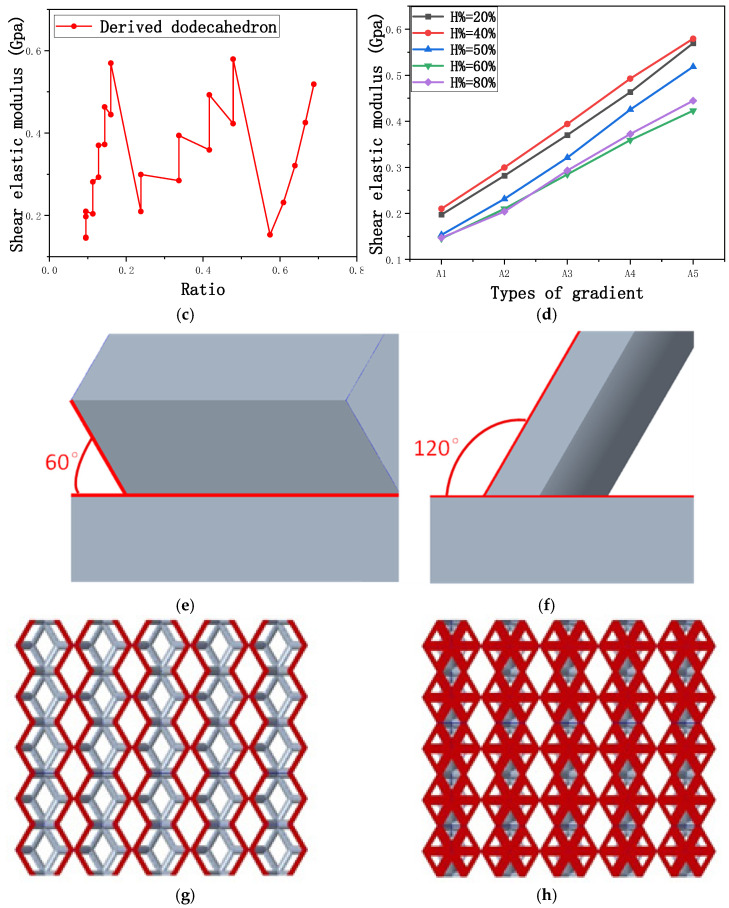
(**a**) Shear elastic modulus of rhombic dodecahedron with respect to Ratio; (**b**) Shear elastic modulus of rhombic dodecahedron with respect to types of gradient of cutting height in the shear direction; (**c**) Shear elastic modulus of derived dodecahedron with respect to ratio; (**d**) Shear elastic modulus of derived dodecahedron with respect to types of gradient of cutting height in the shear direction; (**e**) The angle between the structure of 40% cutting height of rhombic dodecahedron and solid part; (**f**) The angle between the structure of 60% cutting height of rhombic dodecahedron and solid part; (**g**) The contact shape between the solid part and the porous structure of 50% cutting height of the rhombic dodecahedron; (**h**) The contact shape between the solid part and the porous structure of 50% cutting height of the derived dodecahedron.

As shown in Figure 12b,d, the total shear modulus of both models exhibits a linear increase corresponding to changes in the model code, specifically an increase in edge diameter and a decrease in equivalent porosity. The shear modulus curves of the rhombic dodecahedron and its derivative exhibit a similar trend, attributed to the increase in the upper and lower limits of porosity, i.e., the decrease in porosity, which leads to an increase in the shear elastic modulus of the model. As shown in Figure 12a,c, the Ratio and shear modulus of the two models generally show an upward trend, but not linearly, as shown in Figure 12e,f. Due to the symmetrical geometric characteristics of both the rhombic dodecahedron and its derived dodecahedron unit cells, the contact area between the porous structure and the solid part is almost identical when H% is 40% and 60%, respectively. When the model is subjected to external forces, at a unit cell cutting height of 40%, the angle between the model’s strut and the applied force is less than 90°, enabling it to effectively resist deformation. However, at a cutting height of 60%, the angle between the strut and the applied force exceeds 90°, weakening its resistance to external forces. This results in a nonlinear increase in the shear elastic modulus as the ratio increases. The elastic modulus of the fifth-to-last data point experiences a sudden and significant decrease, after which the subsequent points exhibit a linear increasing trend. The vertical symmetry of the model also leads to the maximum contact area at the middle height. When cut at 50% of the height, the contact area is significantly larger compared to other cutting heights, as shown in Figure 12g,h. However, the edge diameter at this height is relatively small, indicating high porosity and weak shear resistance. As the edge diameter increases and porosity decreases, the shear modulus steadily increases, confirming the expected relationship between structural density and shear resistance.

Table 7 presents the stress distribution of two models, with and without gradient, in the shear direction with respect to time, corresponding to the time intervals of 0 s, 0.25 s, 0.5 s, 0.75 s, and 1 s at the end. From the table, it is evident that in both non-gradient models, stress originates from the unit cell based on the solid part and diffuses upwards as time progresses. By the end of the compression, stress concentration occurs at the bottom, with almost no stress at the top. In contrast, the gradient model generates stress from the middle and surrounding areas, which first diffuses downwards and then upwards with respect to time. Using Creo with the specified parameters—density of 4172 kg/m^3^, elastic modulus of 28.78 Gpa, and Poisson’s ratio of 0.3—centroids for both models were calculated, in the middle lower position of the porous structure. The derived dodecahedron experiences force initiation at the centroid, which then spreads outward, reflecting the characteristic gradient model design of good resilience on the outside and a certain degree of rigidity on the inside. This downward diffusion of stress aligns with the original intent of the gradient model, where the low-porosity areas bear the load while the high-porosity areas assist in deformation. For the gradient model of the rhombic dodecahedron, stress initially generates from the top, propagating downward and towards the center. By the end of the compression, there is almost no stress at the bottom or the center of mass, with stress concentration occurring at the top. The key difference in the force transmission mechanism between the two gradient models lies in the alignment of the edge diameters with the direction of force and the coaxial alignment of forces. The derived dodecahedron has more struts aligned with the direction of force, making the stress diffusion at the centroid more pronounced. In comparison, the rhombic dodecahedron lacks struts aligned with the force direction and coaxial direction, which is the main reason for the less efficient stress conduction mechanism in this model. Despite these differences, the stress distribution in Table 7 shows that the gradient model experiences lower overall internal stress compared to the non-gradient model, highlighting the superior mechanical performance of the gradient model. This demonstrates that the gradient structure effectively improves stress distribution and performance under load.

#### 3.2.4. Analysis of Compression Simulation Results for Solid and Porous Structures

**Figure 13:**
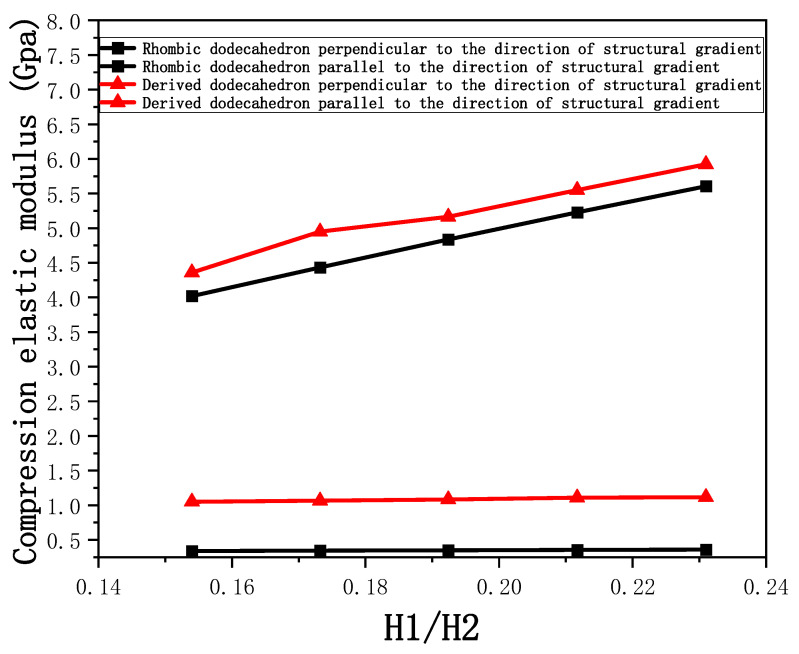
Compression elastic modulus parallel and perpendicular to the direction of the structural gradient of layered structure composed of two types of unit cells with respect to H_1_/H_2_.

As mentioned earlier, the cortical bone attachment range of human long bones is 0.33–3.5 mm. The previous model assumed a solid thickness of 2.5 mm, but the solid thickness would affect the compressive elastic modulus parallel to the direction of structural gradient of layered structure composed of two types of unit cells and perpendicular to the direction of structural gradient of layered structure composed of two types of unit cells. This section discusses the influence and trend of solid thickness on the mechanical properties of porous structures. At the beginning of the design, the modeling cross-sectional area of the solid part was the same as that of the porous structure, so the volume ratio of solid/porous is the ratio of height. In this article, five heights were selected, namely 2 mm, 2.25 mm, 2.5 mm, 2.75 mm, and 3 mm. In order to ensure consistency of variables, the model height of the two porous structures was cut to 0%, which is the initial morphology of the unit cell. The volume ratio of solid/porous mentioned earlier is the ratio of height, so H_1_/H_2_ is defined as the ratio of solid height to porous structure height. That is, the larger H_1_/H_2_, the greater the proportion of solid volume. Two models, identified by the code A_3_, were selected for compression simulations along both parallel and perpendicular to the direction of structural gradient of layered structure composed of two types of unit cells. The boundary conditions applied are consistent with those described earlier, and the corresponding data are presented in Figure 13.

The gradient perpendicular elastic modulus of the rhombic dodecahedron ranges from 4.0187 to 5.6063 Gpa, while the gradient direction elastic modulus ranges from 0.3377 to 0.3603 Gpa. For the derived dodecahedron, the gradient perpendicular elastic modulus ranges from 4.3595 to 5.9238 Gpa, and the gradient direction elastic modulus ranges from 1.0496 to 1.1173 Gpa. It is evident that within the solid thickness range of 2 to 3 mm, the equivalent elastic modulus of both models falls within the range of the humeral elastic modulus. Figure 13 illustrates the relationship between the H_1_/H_2_ ratio and the gradient direction/gradient perpendicular direction elastic moduli for both models. The results show that both the gradient direction and gradient perpendicular direction elastic moduli increase with the increase in the H_1_/H_2_ ratio. However, the increase in the gradient perpendicular direction elastic modulus is more pronounced than that parallel to the direction of structural gradient of layered structure composed of two types of unit cells. This can be attributed to the fact that during compression along perpendicular to the direction of structural gradient of layered structure composed of two types of unit cells, the solid part predominantly bears the load, while the porous part primarily assists in deformation and force transmission. The compressive deformation parallel to the direction of structural gradient of layered structure composed of two types of unit cells is more borne by the porous structure. Since the deformation of the solid part is relatively small, the variation of the elastic modulus is not sensitive to the increase in the thickness of the solid part.

### 3.3. Compressive Mechanical Properties and Failure Analysis

#### 3.3.1. Compressive Mechanical Properties and Morphology Analysis of Gradient Perpendicular Porous Structures

**Figure 14:**
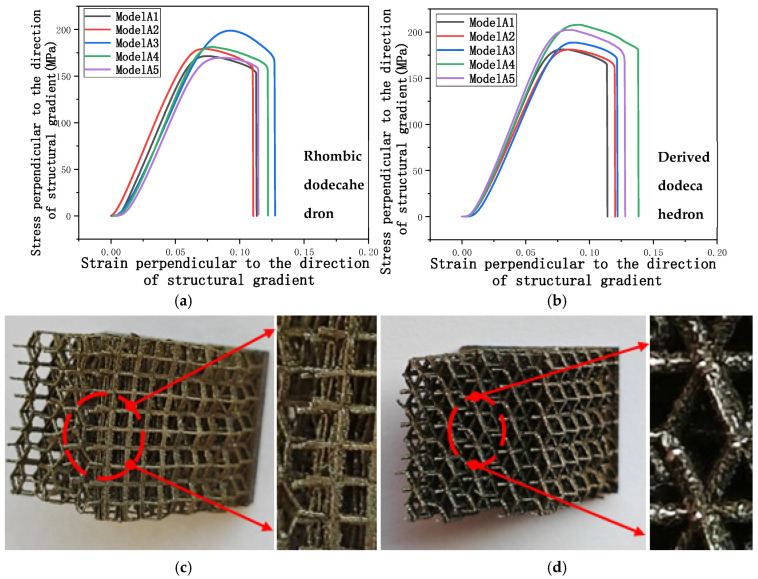

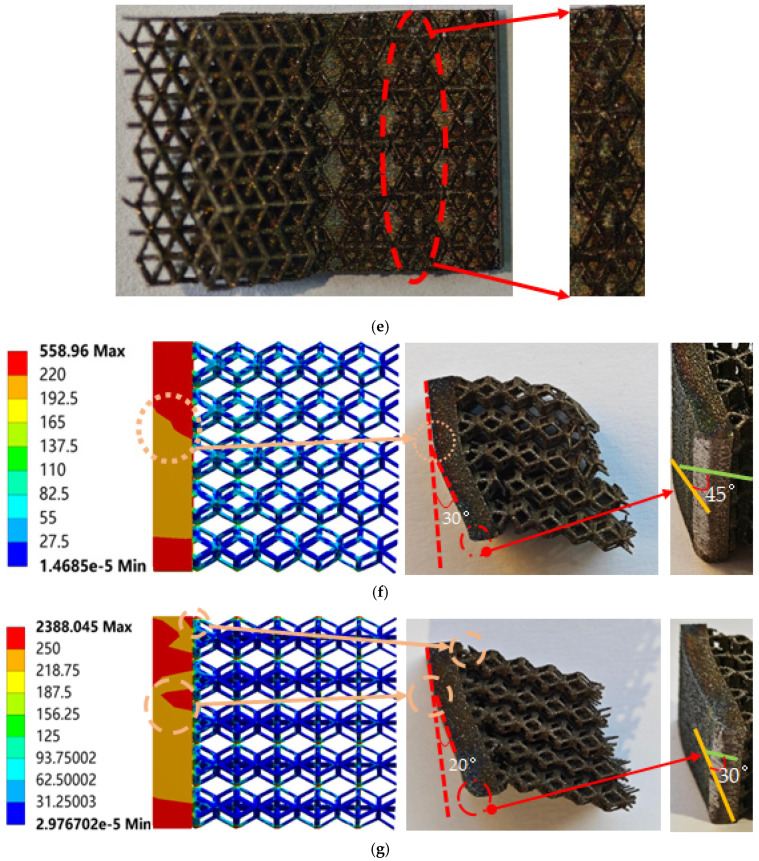
Stress perpendicular to the direction of the structural gradient of layered structure composed of two types of unit cells with respect to strain and the failure of layered structure composed of two types of unit cells in compression perpendicular to the direction of structural gradient. (**a**) Stress perpendicular to the direction of the structural gradient of layered structure composed of rhombic dodecahedron with respect to strain; (**b**) Stress perpendicular to the direction of the structural gradient of layered structure composed of derived dodecahedron with respect to strain; (**c**) The failure of rhombic dodecahedron on the top view; (**d**) The failure of derived dodecahedron on the top view; (**e**) Fracture diagram of rhombic dodecahedron; (**f**) Comparison between simulation and experiment of rhombic dodecahedron; (**g**) Comparison between simulation and experiment of derived dodecahedron.

Through compression experiments, the force displacement curve can be obtained from the experiment, and the stress-strain curve can be calculated based on formulas Equations (2) and (3). Figure 14a,b shows the stress-strain curves of two models compressed perpendicular to the direction of structural gradient of layered structure.

Analyzing these curves provides a deeper understanding of the mechanical properties of the two structural models under compressive loads. In the elastic deformation region of both models, it can be observed that different model codes exhibit similar stress increase patterns and elastic modulus values. This phenomenon occurs because, under compression perpendicular to the gradient direction, the solid part predominantly bears the load and resists compression. The elastic modulus of the gradient porous structure in this direction is significantly smaller than that of the solid structure, with nearly all of the load being carried by the solid structures. Therefore, in the elastic deformation stage, the rhombic dodecahedron models with different gradients show similar elastic modulus values, and the derived dodecahedron follows the same trend. As shown in Figure 14a,b, both the rhombic dodecahedron and its derivative experience sudden fracture after the yielding stage. However, these models exhibit significant ductility before fracture, with strains exceeding 5%, indicating that the overall design possesses the characteristics of ductile materials. Upon failure of both structural models, the strain ranges between 10% and 15%, further confirming that the models are composed of ductile materials [47,48]. During the transition from elastic deformation to plastic deformation, the yield stress magnitudes of the derived dodecahedron follow the order: A_4_ > A_5_ > A_3_ > A_2_ > A_1_. It can be observed that the yield stress is positively correlated with the porosity of the structure: as the edge diameter increases and porosity decreases, the yield stress increases. In contrast, no significant correlation is observed between the yield strength and porosity of the rhombic dodecahedron model. The yield strength values of the rhombic dodecahedron are as follows: A_3_ > A_4_ > A_2_ > A_5_ > A_1_. The speculated reason for this phenomenon is that, during compression, the yield stage of the rhombic dodecahedron model leads to buckling, which increases the shear force within the model, leading to increased shear forces on the model and causing the observed yield strength in the curve to be lower than the actual compressive yield strength.

Figure 14c shows the failure mode of the compressed rhombic dodecahedron porous structure. As clearly shown in the figure, the porous structure has undergone significant deformation, demonstrating considerable ductility. This is attributed to the relatively low porosity of the rhombic dodecahedron. Figure 14d shows the failure mode of the compressed derived dodecahedral porous structure. Compared to the diamond-shaped dodecahedron porous structure in Figure 14c, the deformation of the derived dodecahedron porous structure is relatively small, indicating that the derived dodecahedron has greater rigidity. However, higher strength corresponds to lower ductility.

Figure 14f compares the simulation results of a rhombic dodecahedron with experimental data. As shown in the figure, with the increase in strain, the internal stress distribution within the solid area becomes uneven, leading to buckling in the structure. When the model is initially compressed, the height of the porous structure matches that of the solid structure. During compression, the solid structure, with its higher stiffness and smaller deformation, contrasts with the porous structure, which exhibits lower stiffness and greater deformation. As a result, the model tends to bend to the left, eventually resulting in a 30° offset. The failure observed at the solid bottom is a 45° shear failure, which confirms that the solid structure exhibits characteristics of ductile materials. Figure 14g compares the simulation results of the derived dodecahedron with experimental data. As shown in the figure, the failure mode of the derived dodecahedron is similar to that of the rhombic dodecahedron. However, due to the higher stiffness of the derived dodecahedron’s porous structure compared to the rhombic dodecahedron, the derived dodecahedron exhibits higher maximum internal stress and smaller deformation instability. From a working condition analysis perspective, the structural performance of the derived dodecahedron is not as favorable as that of the rhombic dodecahedron.

Figure 14e shows the failure section of a rhombic dodecahedron with model code A1, which corresponds to the model with the highest porosity. As clearly shown in the figure, the cross-section of the rhombic dodecahedron is not the contact surface between the solid structure and the porous structure, but rather the direct bonding failure of the unit cell. When the solid structure buckles towards the opposite side of the porous structure after instability, the porous structure bears tensile stress. Due to the smaller edge diameter, it is unable to withstand the increased tensile stress, leading to the formation of a cross-section. Only one model in this experiment produced a failure section, while the other models underwent plastic deformation, highlighting the forming advantage of porous structures. In future work, the mechanical strength of the material can be enhanced by increasing the magnesium content. This approach could help prevent structural failure in the model [49].

In compression perpendicular to the gradient direction of the structure, it can be observed that composite structures dominate the compression analysis, while gradient porous structures have a relatively minor impact on the mechanical properties. The performance of the composite structure in compression aligns well with the biomimetic bone working conditions.

#### 3.3.2. Compressive Mechanical Properties and Morphology Analysis of Gradient Parallel Porous Structures

**Figure 15:**
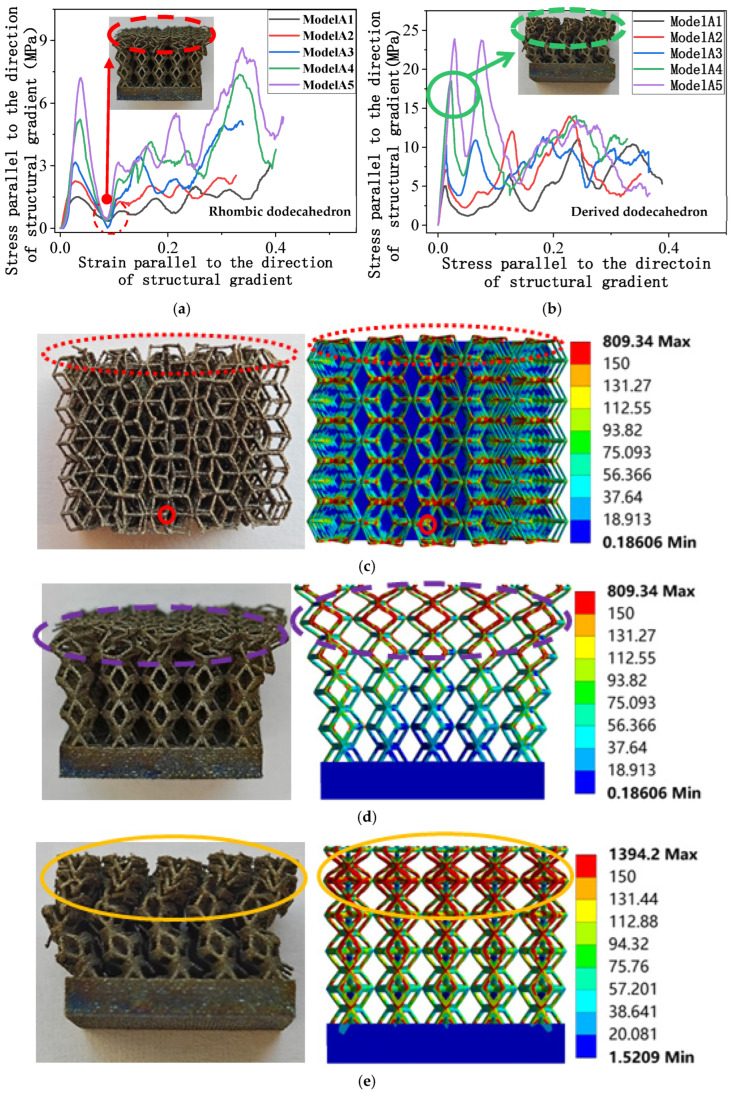
Stress parallel to the direction of the structural gradient of layered structure composed of two types of unit cells with respect to strain and the failure of two structures in compression parallel to the direction of structural gradient of layered structure composed of two types of unit cells. (**a**) Stress parallel to the direction of the structural gradient of layered structure composed of rhombic dodecahedron with respect to strain; (**b**) Stress parallel to the direction of the structural gradient of layered structure composed of derived dodecahedron with respect to strain; (**c**) Comparison between simulation and experiment on the top view of rhombic dodecahedron; (**d**) Comparison between simulation and experiment on the front view of rhombic dodecahedron; (**e**) Comparison between simulation and experiment on the front view of derived dodecahedron.

Figure 15a,b presents the stress-strain curves of two models under compressive stress applied parallel to the direction of structural gradient of layered structure composed of two types of unit cells. In contrast to compression perpendicular to the gradient direction, deformation is primarily accommodated by the porous structures with higher porosity. Both the rhombic dodecahedron and its derivative structures are classified as rod-dominant element configurations. Compared to the triply-periodic minimum curved surface element structure, their stress-strain curves exhibit an alternating pattern of peaks and valleys. Figure 15a shows the stress-strain curve under compression parallel to the direction of structural gradient of layered structure composed of rhombic dodecahedron. It is evident that after undergoing elastic deformation, the stress in the model experiences a sharp decrease. Following a brief stress drop, the stress begins to rise again as the subsequent layers of the model begin to contribute to the load-bearing capacity.

In Figure 15a, the stress of different models of the rhombic dodecahedron structure will decrease to the same range when the first layer fails. This can be attributed to the lack of effective support once the previous structural layer has collapsed. As the next layer begins to participate in bearing the stress, the stress does not approach zero again. Instead, compaction of the first layer after its collapse results in a direct and gradual increase in the overall stress of the structure.

Figure 15b shows the stress-strain curve parallel to the direction of structural gradient of layered structure composed of derived dodecahedron. It can be seen that the trend of the derived dodecahedron is similar to that of the rhombic dodecahedron. After experiencing elastic deformation, the stress decreases due to structural failure, and the stress increases after the intervention of the next layer structure. But the second peak of the derived dodecahedron is almost identical to the first peak, and as the compression continues, the stress increases. In the circle, it can be clearly seen that in the first two layers of the structure, the failure direction of the model is along the structural gradient of layered structure composed of two types of unit cells. When the third layer begins to fail, the model shows a buckling, and the direction of instability is random. This is because the derived dodecahedron internal truss leads to the overall rigidity of the structure, making it more prone to instability after compression. The yield strength of the derived dodecahedron model is the same as that of the rhombic dodecahedron, that is, the stress peak at the same strain increases with the decrease of porosity.

Figure 15c,d show the top and front views of the simulation and experimental comparison of a rhombic dodecahedron. It can be seen from the figures that the stress concentration observed in the simulation resulted in varying degrees of failure in the experiment, which confirms the consistency between the simulation and experimental results.

Figure 15e presents the front view of the derived dodecahedron, comparing the simulation results with the experimental data. As shown, stress concentration in the simulation occurs at the top of the structure, which corresponds to the failure mode observed in the experimental results. This consistency between the simulation and experimental findings further validates the accuracy of the simulation model. In compression parallel to the direction of the structural gradient of layered structure composed of two types of unit cells, it can be observed that layered porous structures dominate the analysis of compression results, while composite structures have a relatively small impact on the analysis of mechanical properties.

#### 3.3.3. The Influence of Solid Part Thickness of Composite Structures on Compressive Mechanical Properties Perpendicular to the Direction of Structural Gradient of Layered Structure Composed of Two Types of Unit Cells

**Figure 16:**
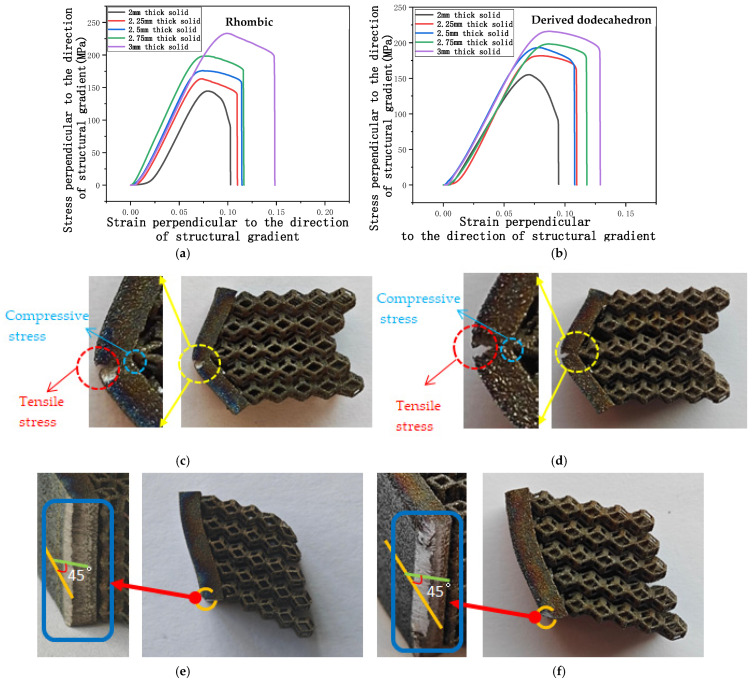
Stress perpendicular to the direction of the structural gradient of layered structure composed of two types of unit cells with respect to strain of solid thickness and the failure of two unit cells in compression perpendicular to the direction of structural gradient of layered structure composed of two types of unit cells. (**a**) Stress perpendicular to the direction of the structural gradient of layered structure composed of rhombic dodecahedron with respect to strain of solid thickness; (**b**) Stress perpendicular to the direction of the structural gradient of layered structure composed of derived dodecahedron with respect to strain of solid thickness; (**c**) The failure of rhombic dodecahedron with the solid thickness of 2 mm on the front view; (**d**) The failure of derived dodecahedron with the solid thickness of 2 mm on the front view; (**e**) The failure of rhombic dodecahedron with the solid thickness over 2 mm on the front view; (**f**) The failure of rhombic dodecahedron with the solid thickness over 2 mm on the front view.

Figure 16 shows the stress-strain curves of the two models as the solid thickness increases. The curves for both models exhibit trends similar to those presented in Section 3.3.1. The stress-strain curves for the rhombic dodecahedron and its derivative dodecahedron are shown in Figure 16a and Figure 16b, respectively. From the figure, it is evident that the yield strength of the model is directly proportional to the solid thickness, meaning that as the thickness of the solid part increases, the yield strength also increases.

Figure 16c,d show the cross-sectional structures of the two models with a solid thickness of 2 mm. From the images, it is apparent that the model does not fail because of shear stress. From the images, it is apparent that the model does not fail because of shear stress. From the figure, it can be seen that the outer side of the solid part is subjected to tensile stress and the inner side is subjected to compressive stress, both of which belong to normal stress. However, as the thickness increases, failure shifts to being dominated by shear stress, as shown in Figure 16e,f. Under compressive loading, thinner metal plates typically exhibit a more uneven stress distribution, where normal stress is more likely to induce failure, especially when the material enters the yield or plastic deformation state. As the thickness increases, the influence of shear stress becomes more pronounced. This is due to the creation of a stronger shear stress gradient during the transmission of compressive forces, particularly near the contact surfaces. In this scenario, the material’s shear strength becomes more critical than its yield strength from normal stress, resulting in a shift in the failure mode from normal stress-induced yielding or crushing to shear stress-induced failure.

### 3.4. Comparison Between Simulation and Experiment

**Figure 17:**
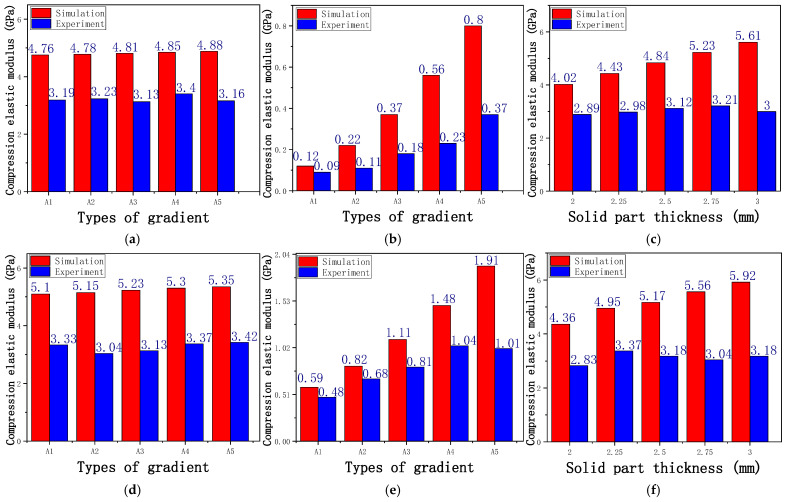
Bar chart of actual elastic modulus and simulated elastic modulus of rhombic dodecahedron and derived dodecahedron; (**a**) Rhombic dodecahedron perpendicular to the direction of structural gradient compression; (**b**) Rhombic dodecahedron parallel to the direction of structural gradient compression; (**c**) Rhombic dodecahedron solid part thickness variation; (**d**) Derived dodecahedral perpendicular to the direction of structural gradient; (**e**) Derived dodecahedral parallel to the direction of structural gradient; (**f**) Derived dodecahedron solid part thickness variation.

The yield strength of the rhombic dodecahedron perpendicular to the direction of structural gradient compression ranges from 157.5609 to 179.9329 MPa, with an elastic modulus between 3.1299 and 3.3964 GPa. In parallel to the direction of structural gradient of layered structure, the compressive yield strength is between 1.1454 and 7.1243 MPa, and the elastic modulus ranges from 0.0871 to 0.3671 GPa. As the solid thickness of the model increases from 2 mm to 3 mm, the yield strength of the rhombic dodecahedron perpendicular to the direction of structural gradient of layered structure increases to between 127.0583 and 198.0938 MPa, with an elastic modulus ranging from 2.8962 to 3.3013 GPa. The actual measured elastic modulus is lower than the theoretical range, which is between 0.1 and 17.5 GPa. A bar chart comparing the simulated and actual elastic modulus values is shown in Figure 17. The comparison data between simulation and experiment are shown in Table 8, it can be seen that both the comparison between simulation and experimental data, as well as the maximum yield strength under the three loading conditions, indicate that the elastic modulus and maximum yield strength of the model increase with types of gradient.

From Figure 17a–c, it can be observed that the simulated elastic modulus is consistently higher than the actual measured elastic moduli. This discrepancy can be attributed to several factors: (1) The simulated operating conditions are idealized, eliminating potential issues such as model instability and deviations in force direction; (2) During the simulation, only normal stress is considered, while in experiments, material failures predominantly occur under shear stress, similar to issues related to instability; (3) The actual porosity obtained is higher than the designed porosity due to the nature of the SLM (Selective Laser Melting) process. As shown in Figure 17a, the measured elastic modulus remains relatively unchanged during compression in perpendicular to the direction of structural gradient of layered structure composed of rhombic dodecahedron. This is because the overall structure is solid under stress, and the hollow sections contribute minimally to the force-bearing capacity. Furthermore, the high porosity and ductility of the rhombic dodecahedron result in almost no variation in the actual elastic modulus. In contrast, for compression parallel to the direction of structural gradient of layered structure composed of rhombic dodecahedron (Figure 17b), the elastic modulus exhibits a clear upward trend with an increase in edge diameter and a decrease in porosity, consistent with the simulation results. This demonstrates the advantages of SLM technology in printing gradient models. As the solid thickness increases, the elastic modulus fluctuates within a certain range due to the model’s thin-sheet structure, which also experiences shear stress (Figure 17c). This fluctuation is the primary manifestation of the model’s stability during compression.

## 4. Conclusions

This article discusses the preparation of layered gradient composite structures based on rhombic dodecahedron and derived dodecahedron unit cells using SLM technology. By applying the Ashby-Gibson theory, the porosity of the porous structure was controlled within the range of 74.45% to 93.52%, achieving a macroscopic elastic modulus for the titanium alloy porous structure biomimetic bone in the range of 0.1 to 17.5 GPa, thereby reducing the stress shielding effect. When combined with the solid structure, the composite structure achieved biomimetic matching of both mechanical properties and bone tissue structure.

Under compression conditions parallel to the gradient direction of the model structure, the elastic modulus of the rhombic dodecahedron ranges from 0.1131 to 0.5127 GPa, while that of the derived dodecahedron ranges from 0.3827 to 2.3107 GPa. Additionally, compared to the non-gradient structure model, the gradient structure in both models has a more significant impact on regulating the direction of force transmission. Under compression conditions perpendicular to the gradient direction of the structure, the elastic modulus of the rhombic dodecahedron ranges from 4.7636 to 4.9101 GPa, while that of the derived dodecahedron ranges from 5.0531 to 5.4365 GPa. Compared to non-gradient structural models, the gradient models of both structures fully engage in stress distribution along the struts parallel to the direction of the structural gradient of layered structure composed of two types of unit cells, resulting in a more uniform and reasonable stress distribution. Under the shear direction conditions of the model, the elastic modulus of the rhombic dodecahedron is 0.033–0.3876 GPa, and the derived dodecahedron is 0.1445–0.5794 GPa. The internal stress of the derived dodecahedron gradient structure appears from the center of mass and diffuses outward; this results in the model exhibiting characteristics of good resilience on the outside and a certain degree of rigidity on the inside. The internal stress mechanism of the rhombic dodecahedron is the opposite, with stress gradually transmitted from the top edge downwards and towards the center. Compared to the non-gradient model, the gradient model has lower overall internal stress and exhibits superior mechanical performance.

The structural and mechanical advantages of gradient structures in the field of biomimetic bone make them have enormous clinical application potential in the future multi-dimensional, multi-scale, and highly customized gradient structure biomimetic bone field. Meanwhile, this study provides new insights into the force transmission mechanism of gradient structures and the relationship between the direction of structural struts, which can be further quantified in the future. Meanwhile, the use of hierarchical gradients also provides a theoretical foundation for highly personalized customization. Further exploration is needed to investigate the bone growth and osseointegration mechanisms towards alloy porous structures, as well as the surface modification of porous structures.

## Figures and Tables

**Table 1:** The contact shape between the cut model and the solid part.

	Model	Unit Cell of Rhombic Dodecahedron	Unit Cell of Derived Dodecahedron	Rhombic Dodecahedron	Derived Dodecahedron
H%					
**20%**		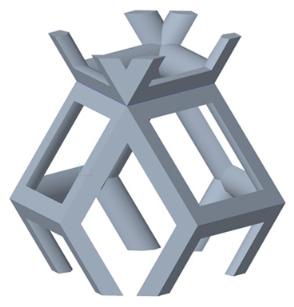	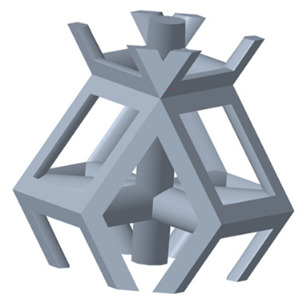	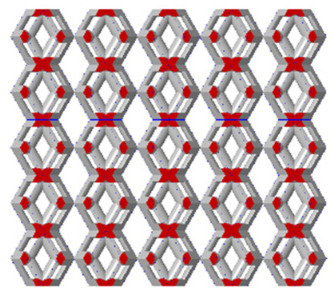	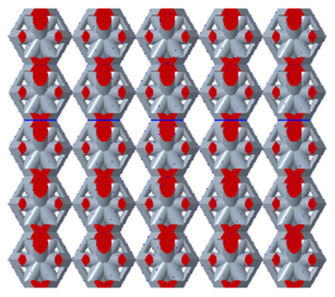
**40%**		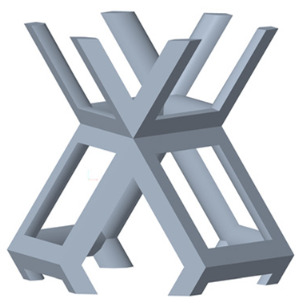	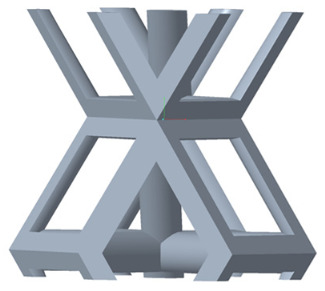	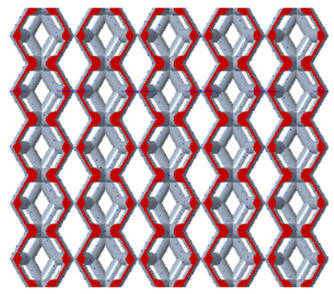	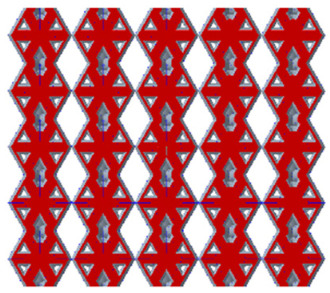
**50%**		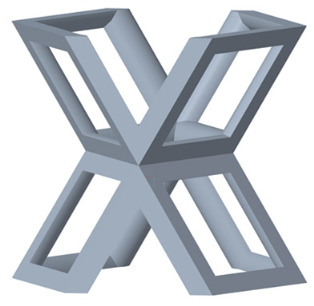	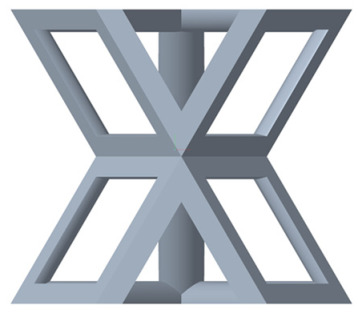	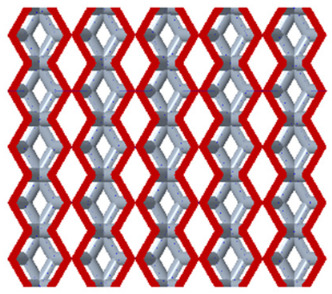	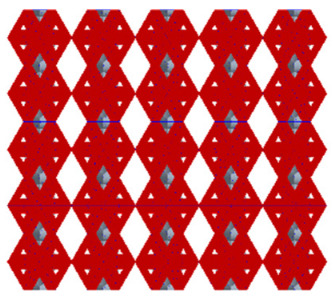
**60%**		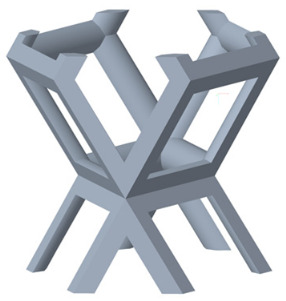	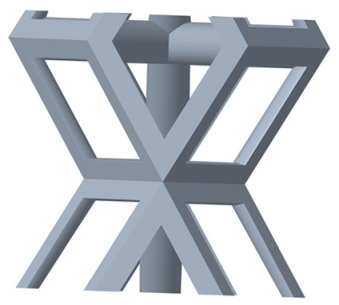	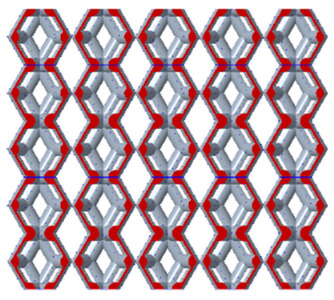	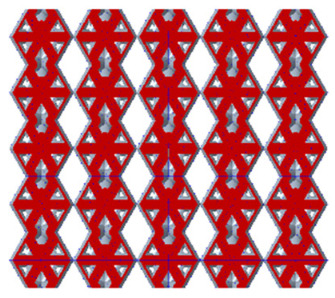
**80%**		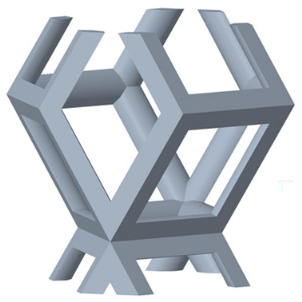	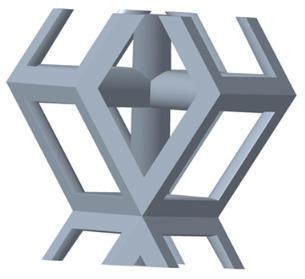	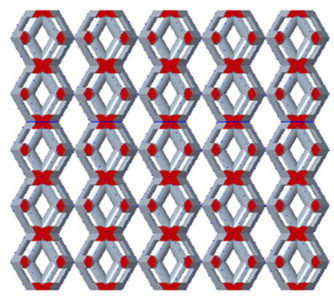	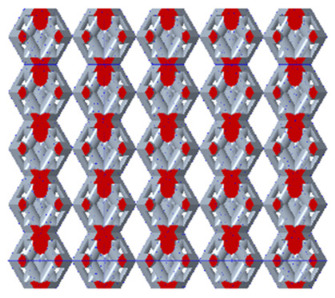

**Table 2:** Chemical composition of Ti6Al4V powder.

Element	Ti	Al	V	Fe	C	N	H	O
Content, %	Other	6.46	4.04	0.22	0.011	0.01	0.002	0.078

**Table 3:** Two views of different cutting height of two unit cells.

Unit Cell		H%	20%	40%	50%	60%	80%
	Angle of View						
**Rhombic Dodecahe-dron**	**Top** **view**		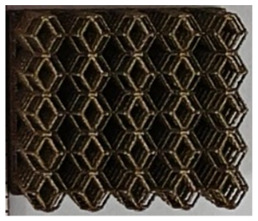	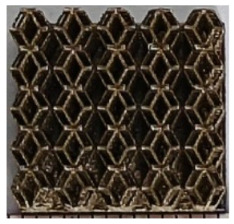	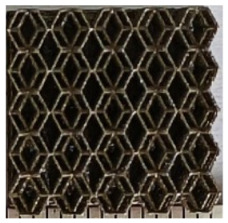	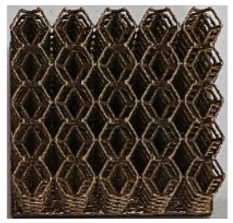	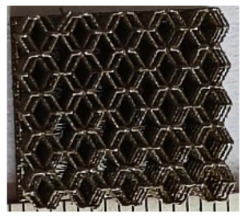
	**Front** **view**		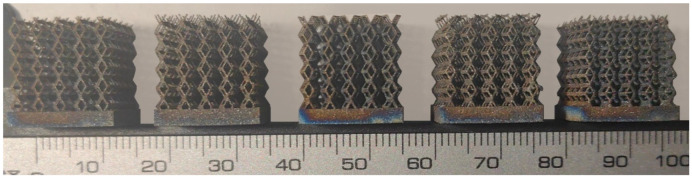				
**Derived** **Dodecahe-dron**	**Top** **view**		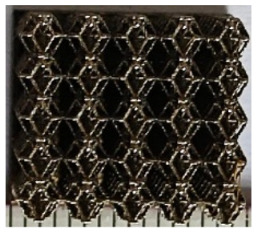	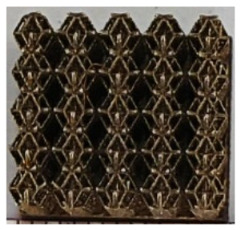	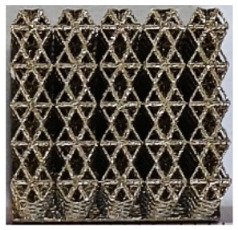	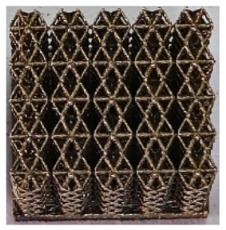	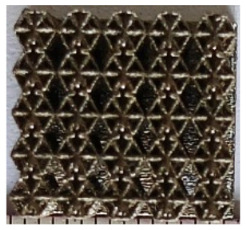
	**Front** **view**		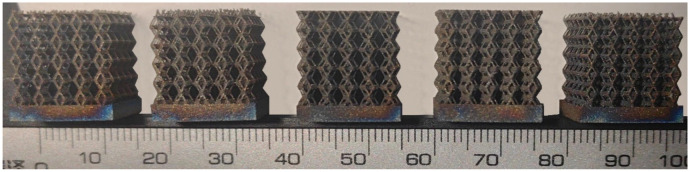				

**Table 4:** Stress distributions of gradient composite structures and non-gradient composite structures under compressive forces parallel to the gradient direction.

	Stress Distribution			T = 0 s	T = 0.25 s	T = 0.5 s	T = 0.75 s	T = 1 s
Rhombic Dodecahedron		Non-gradient	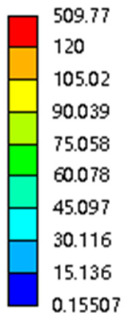	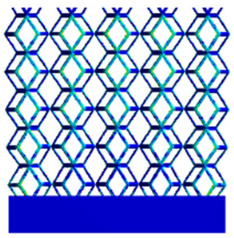	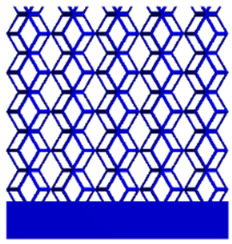	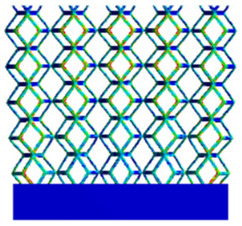	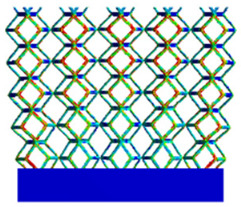	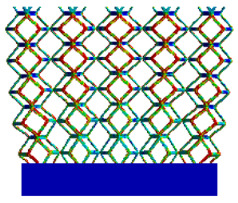
		Gradient	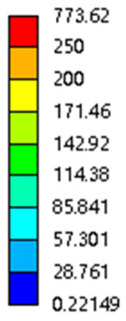	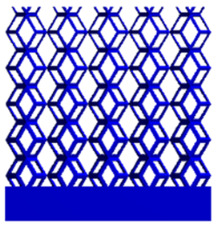	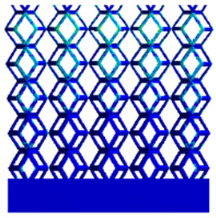	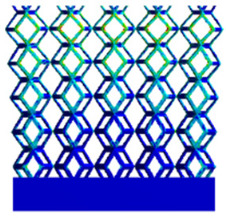	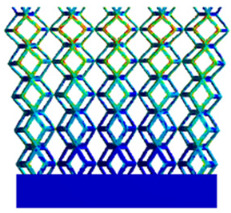	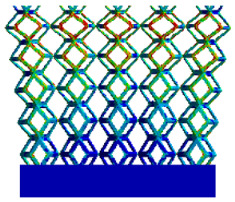
Derived Dodecahedron		Non-gradient	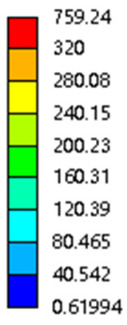	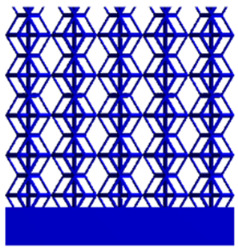	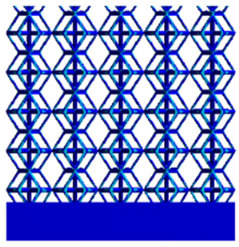	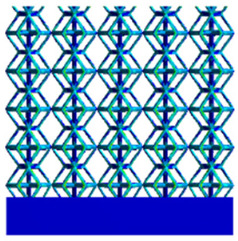	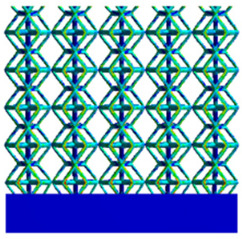	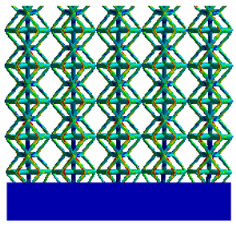
		Gradient	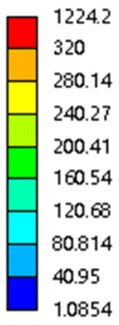	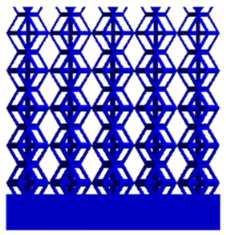	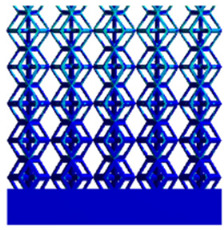	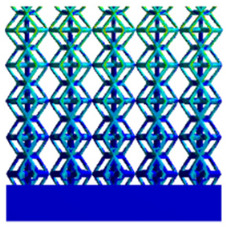	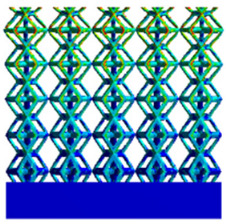	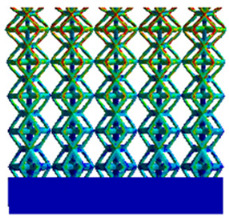

**Table 5:** Stress distributions of gradient composite structures and non-gradient composite structures under compressive force perpendicular to the gradient direction.

	Stress Distribution		T = 0 s	T = 0.25 s	T = 0.5 s	T = 0.75 s	T = 1 s
Rhombic Dodecahedron	Non-gradient	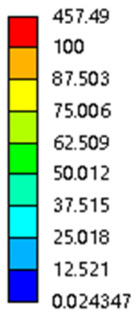	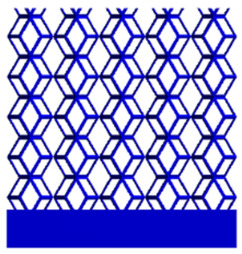	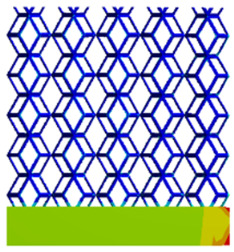	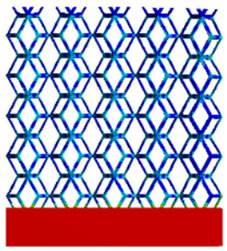	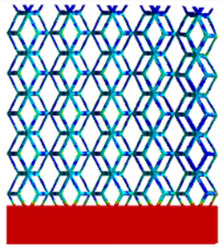	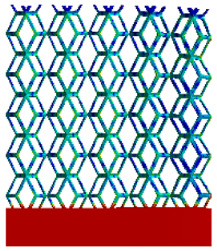
	Gradient	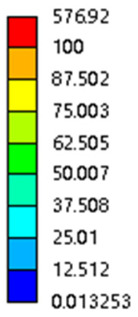	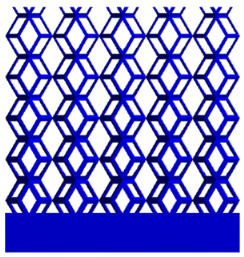	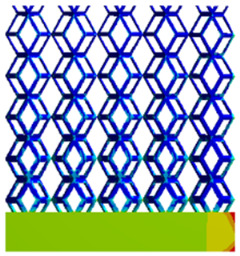	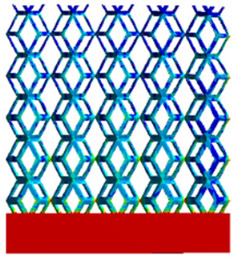	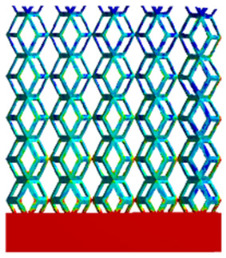	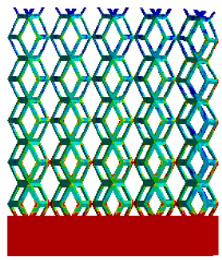
Derived Dodecahedron	Non-gradient	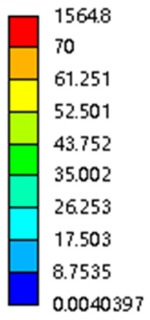	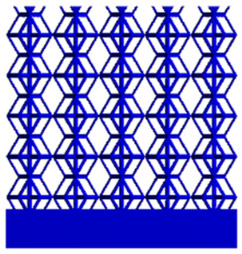	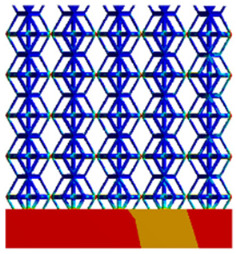	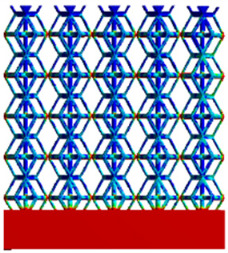	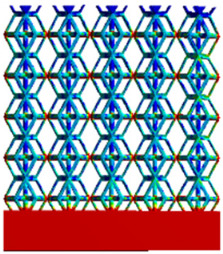	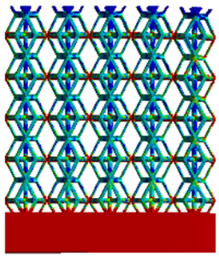
Derived Dodecahedron	Gradient	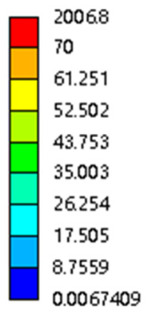	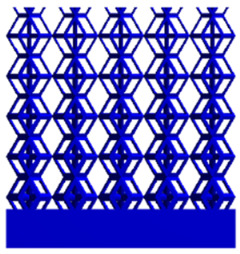	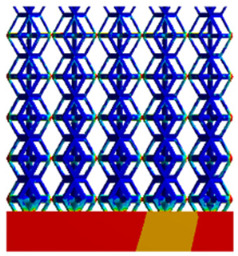	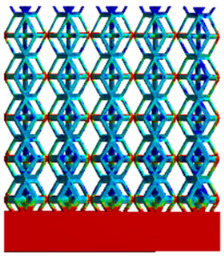	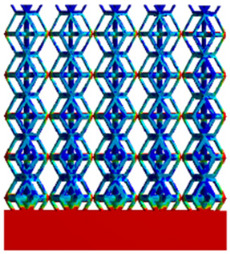	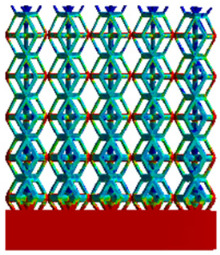

**Table 6:** Shear elastic modulus and ratio of two models in the shear direction.

**Shear elastic modulus G (Gpa) and ratio of rhombic dodecahedron**										
	20%		40%		50%		60%		80%	
	ratio	G	ratio	G	ratio	G	ratio	G	ratio	G
**A_1_**	0.059	0.0651	0.059	0.0677	0.197	0.0841	0.059	0.033	0.059	0.0416
**A_2_**	0.071	0.1078	0.071	0.1105	0.217	0.1438	0.071	0.0557	0.071	0.0709
**A_3_**	0.082	0.1627	0.085	0.1546	0.237	0.2237	0.085	0.088	0.082	0.1125
**A_4_**	0.094	0.2294	0.128	0.255	0.257	0.308	0.128	0.1282	0.094	0.1653
**A_5_**	0.107	0.2844	0.159	0.3093	0.278	0.3876	0.159	0.171	0.107	0.2196
**Shear elastic modulus G (Gpa) and ratio of derived dodecahedron**										
	20%		40%		50%		60%		80%	
	ratio	G	ratio	G	ratio	E	ratio	E	ratio	E
**A_1_**	0.095	0.1972	0.095	0.2101	0.574	0.1533	0.095	0.1454	0.095	0.1471
**A_2_**	0.113	0.2817	0.238	0.2995	0.609	0.2315	0.238	0.2098	0.113	0.204
**A_3_**	0.128	0.3702	0.337	0.3941	0.639	0.3211	0.337	0.2847	0.128	0.2931
**A_4_**	0.144	0.4634	0.416	0.4927	0.666	0.4255	0.416	0.3592	0.144	0.3723
**A_5_**	0.160	0.5696	0.478	0.5794	0.688	0.5185	0.478	0.4229	0.160	0.4448

**Table 7:** Stress distributions of gradient composite structures and non-gradient composite structures under shear force applied at the interface between the solid part and porous part.

	Stress Distribution		T = 0 s	T = 0.25 s	T = 0.5 s	T = 0.75 s	T = 1 s
Rhombic Dodecahedron	Non-gradient	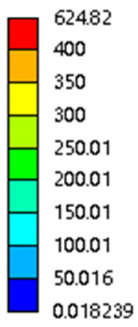	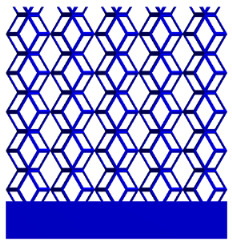	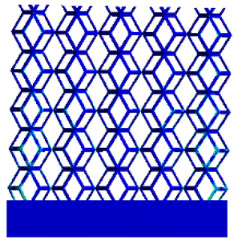	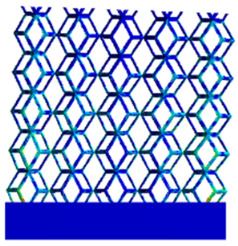	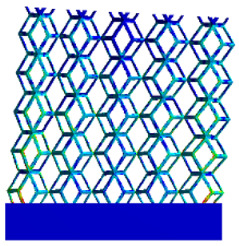	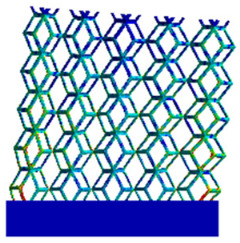
	Gradient	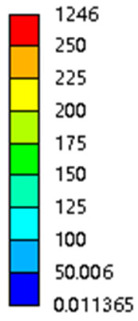	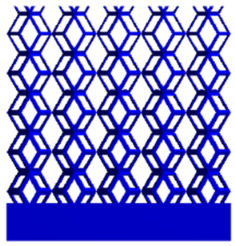	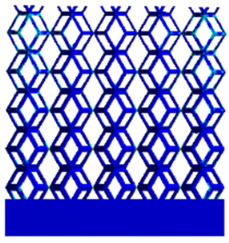	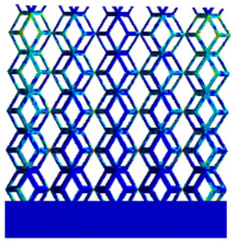	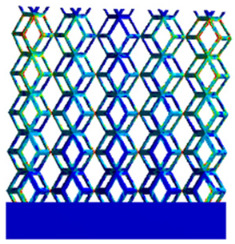	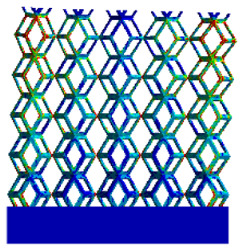
Derived Dodecahedron	Non-gradient	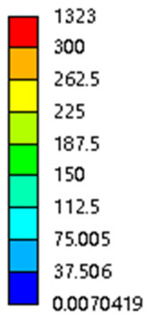	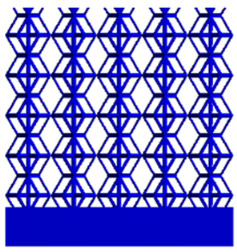	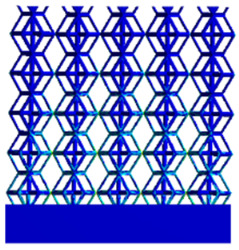	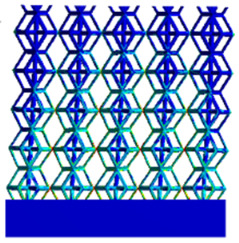	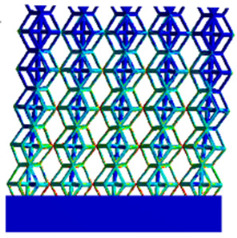	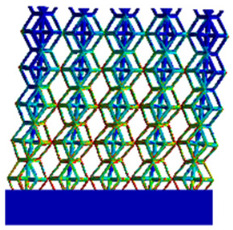
	Gradient	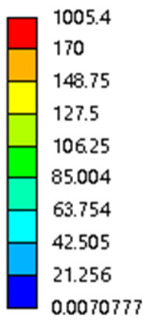	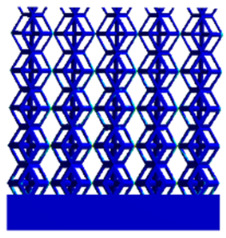	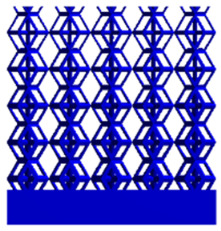	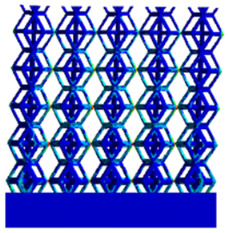	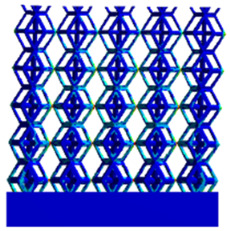	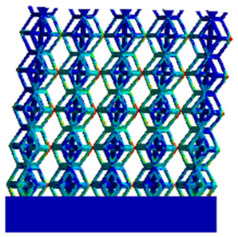

**Table 8:** Comparison data between simulation and experiment.

**Rhombic Dodecahedron**											
		Compression perpendicular to the direction of the structural gradient (GPa)		Compression parallel to the direction of the structural gradient (GPa)		Yield strength (MPa)	Compression with the solid thickness (GPa)				Yield strength (MPa)
	Condition	simulation	experiment	simulation	experiment	perpendicular to the direction of the structural gradient		Condition	simulation	experiment	with the solid thickness
Types of gradient							Thickness				
A_1_		4.7636	3.1944	0.1248	0.087	171.4489	2 mm		4.0187	2.8926	144.4197
A_2_		4.7831	3.2347	0.2218	0.1145	179.2366	2.25 mm		4.4342	2.9753	163.4112
A_3_		4.8086	3.1299	0.367	0.1768	198.7276	2.5 mm		4.8369	3.1198	176.0849
A_4_		4.845	3.3964	0.5642	0.2323	181.3885	2.75 mm		5.2282	3.2039	198.3723
A_5_		4.8796	3.1634	0.8036	0.3671	169.7109	3 mm		5.6063	3.0013	233.1517
**Derived dodecahedron**											
	Condition	simulation	experiment	simulation	experiment	perpendicular to the direction of the structural gradient		Condition	simulation	experiment	with the solid thickness
Types of gradient							Thickness				
A_1_		5.116	3.3310	0.5787	0.4821	181.0333	2 mm		4.3595	2.8258	155.1019
A_2_		5.1505	3.0442	0.8175	0.6844	181.0889	2.25 mm		4.9503	3.3677	181.7916
A_3_		5.2306	3.1326	1.1163	0.8133	188.5991	2.5 mm		5.1655	3.1793	193.0700
A_4_		5.2974	3.3734	1.4791	1.0435	207.9181	2.75 mm		5.5512	3.0440	198.3165
A_5_		5.3497	3.4220	1.9034	1.0122	202.2822	3 mm		5.9238	3.1758	216.0684

## Data Availability

The original contributions presented in this study are included in the article. Further inquiries can be directed to the corresponding author.
